# Age and Gender Variations in Cancer Diagnostic Intervals in 15 Cancers: Analysis of Data from the UK Clinical Practice Research Datalink

**DOI:** 10.1371/journal.pone.0127717

**Published:** 2015-05-15

**Authors:** Nafees U. Din, Obioha C. Ukoumunne, Greg Rubin, William Hamilton, Ben Carter, Sal Stapley, Richard D. Neal

**Affiliations:** 1 North Wales Centre for Primary Care Research, College of Health and Behavioural Sciences, Bangor University, Wrexham, United Kingdom; 2 NIHR CLAHRC South West Peninsula, University of Exeter Medical School, Exeter, United Kingdom; 3 School of Medicine, Pharmacy and Health, Wolfson Research Institute, Durham University, Durham, United Kingdom; 4 University of Exeter Medical School, Exeter, United Kingdom; 5 Institute of Primary Care & Public Health, Cardiff School of Medicine, Cardiff University, Cardiff, United Kingdom; National Cancer Center, JAPAN

## Abstract

**Background:**

Time from symptomatic presentation to cancer diagnosis (diagnostic interval) is an important, and modifiable, part of the patient’s cancer pathway, and can be affected by various factors such as age, gender and type of presenting symptoms. The aim of this study was to quantify the relationships of diagnostic interval with these variables in 15 cancers diagnosed between 2007 and 2010 using routinely collected data from the Clinical Practice Research Datalink (CPRD) in the UK.

**Methods:**

Symptom lists for each cancer were prepared from the literature and by consensus amongst the clinician researchers, which were then categorised into either *NICE* qualifying (*NICE*) or not (*non-NICE*) based on *NICE* Urgent Referral Guidelines for Suspected Cancer criteria. Multivariable linear regression models were fitted to examine the relationship between diagnostic interval (outcome) and the predictors: age, gender and symptom type.

**Results:**

18,618 newly diagnosed cancer patients aged ≥40 who had a recorded symptom in the preceding year were included in the analysis. Mean diagnostic interval was greater for older patients in four disease sites (difference in days per 10 year increase in age; 95% CI): bladder (10.3; 5.5 to 15.1; P<0.001), kidney (11.0; 3.4 to 18.6; P=0.004), leukaemia (18.5; 8.8 to 28.1; P<0.001) and lung (10.1; 6.7 to 13.4; P<0.001). There was also evidence of longer diagnostic interval in older patients with colorectal cancer (P<0.001). However, we found that mean diagnostic interval was shorter with increasing age in two cancers: gastric (-5.9; -11.7 to -0.2; P=0.04) and pancreatic (-6.0; -11.2 to -0.7; P=0.03). Diagnostic interval was longer for females in six of the gender non-specific cancers (mean difference in days; 95% CI): bladder (12.2; 0.8 to 23.6; P=0.04), colorectal (10.4; 4.3 to 16.5; P=0.001), gastric (14.3; 1.1 to 27.6; P=0.03), head and neck (31.3; 6.2 to 56.5; P=0.02), lung (8.0; 1.2 to 14.9; P=0.02), and lymphoma (19.2; 3.8 to 34.7; P=0.01). Evidence of longer diagnostic interval was found for patients presenting with *non-NICE* symptoms in 10 of 15 cancers (mean difference in days; 95% CI): bladder (62.9; 48.7 to 77.2; P<0.001), breast (115.1; 105.9 to 124.3; P<0.001), cervical (60.3; 31.6 to 89.0; P<0.001), colorectal (25.8; 19.6 to 31.9; P<0.001), gastric (24.1; 3.4 to 44.8; P=0.02), kidney (22.1; 4.5 to 39.7; P=0.01), oesophageal (67.0; 42.1 to 92.0; P<0.001), pancreatic (48.6; 28.1 to 69.1; P<0.001), testicular (36.7; 17.0 to 56.4; P< 0.001), and endometrial (73.8; 60.3 to 87.3; P<0.001). Pooled analysis across all cancers demonstrated highly significant evidence of differences overall showing longer diagnostic intervals with increasing age (7.8 days; 6.4 to 9.1; P<0.001); for females (8.9 days; 5.5 to 12.2; P<0.001); and in *non-NICE* symptoms (27.7 days; 23.9 to 31.5; P<0.001).

**Conclusions:**

We found age and gender-specific inequalities in time to diagnosis for some but not all cancer sites studied. Whilst these need further explanation, these findings can inform the development and evaluation of interventions intended to achieve timely diagnosis and improved cancer outcomes, such as to provide equity across all age and gender groupings.

## Introduction

Rapid diagnosis of cancer after symptoms arise is believed to be important to improve outcomes [1], and patient and/or their carer experience [2, 3]. It is thought that thousands of deaths may be avoided annually if cancers are diagnosed quickly and successfully treated [4–7]. Hence, prompt diagnosis of symptomatic patients has become a priority worldwide [1, 7–9]. The National Awareness and Early Diagnosis Initiative (NAEDI) in England [10] and similar initiatives elsewhere in Europe [9] are trying to address this.

Most patients with cancer-related symptoms present to a primary health care practitioner, usually a GP, who then has to suspect a cancer, or other illness, and initiate an investigation or referral for diagnosis. This period between the first primary care presentation of potential cancer symptoms and eventual diagnosis, the ‘diagnostic interval’ [11, 12] is one of the important phases in the route to diagnosis of many cancers [11, 13]. Shorter diagnostic interval is generally considered to contribute to overall earlier stage diagnoses and better cancer outcomes [5, 6]. Suspecting a cancer diagnosis in primary care may be difficult, as many of the symptoms of cancer can arise from co-morbidities or benign causes [14]. Hence, there is both a potential for delay at this point [12, 13], as well as an opportunity to detect a cancer earlier [15], as an estimated one in 20 consultations in primary care include possible malignant symptomatology [16]. The speed of cancer diagnosis may vary by demographic characteristics, such as age and gender, [17, 18] making some groups vulnerable and disadvantaged in both being diagnosed and treated late, [19–21], leading to poorer survival [22].

Primary care datasets are a key resource for studying cancer diagnostic pathways and have previously been used to determine the positive predictive value of cancer symptoms [23, 24]; the change in diagnostic interval over time for various cancers [12]; and to construct clinical decision support tools [25, 26]. These datasets can also be used to examine the association between the time to diagnosis and demographic variables for specific cancer symptoms presented to primary care.

The aim of this study was to quantify differences in cancer diagnostic intervals across subgroups defined by age, gender and symptom type in 15 types of incident cancer diagnosed between 2007 and 2010 in England and Wales, UK using routinely collected primary care data. This could facilitate an understanding of variation in diagnostic interval and inform the development and evaluation of targeted interventions to facilitate timelier diagnosis.

## Methods

This analysis was undertaken alongside a previously reported study [12], extending the scope to examining the relationship of diagnostic interval with age, gender and symptom type. A more detailed description of applying cancer (S1 Table) and symptom (S2 and S3 Tables) codes to the dataset, and the process of identification and validation of these codes is given in that report and has been supplied as supplementary files for readers’ reference for this report.

Ethical approval for this study was obtained from the Independent Scientific Advisory Committee (ISAC), under license numbers 09_0110 and 09_0111. All patient records/information was anonymised and de-identified when the dataset was obtained from the Clinical Practice Research Datalink—CPRD (General Practice Research Database-GPRD, at the time the data was acquired) and the analysis did not comprise any patient identifiable data.

### Source population dataset

We used routinely collected UK general practice data obtained from the CPRD for 15 types of incident cancer (bladder, breast, cervical, colorectal, endometrial, gastric, head & neck, kidney, lung, leukaemia, lymphoma, myeloma, oesophageal, pancreatic, testicular) with at least one year of complete records before diagnosis. The CPRD is a large, longitudinal general practice database holding anonymised records of over five million active patients registered with over 650 general practices in England and Wales in the UK. General practices that agree to and fulfil strict quality criteria for data entry and maintenance only can contribute to this database and the data is then periodically quality checked to ascertain and maintain its robustness. At the practice level, the GP enters the most appropriate terms related to symptoms or diagnosis based on a list of drop down choices corresponding to the appropriate Oxford Medical Information Systems (OXMIS) and Read codes [27].

The dataset used in this study consisted of patients aged ≥40 years diagnosed between 1^st^ Jan 2007 and 31^st^ Dec 2010 inclusive with one of 15 cancers of interest described earlier.

### *NICE* cancer symptom categories (*NICE* status)

Lists of potential symptoms (S4 Table) of primary, local and regional disease for the cancers of interest for this study were developed from the literature, and by consensus, amongst the three clinician researchers (RN, WH, GR), and were classified into ‘*NICE*-qualifying symptoms’ (*NICE*) or not (*non-NICE*) [12] (S5 Table). These symptom categories are sometimes referred to as ‘alarm symptoms’ and ‘vague symptoms’ respectively in the literature [23]. *NICE* symptoms were those specifically cited in the *NICE* Guideline for Urgent Referral of Suspected Cancer [28] as mandating urgent investigation or specialist assessment.

### Diagnostic interval

The first occurrence of a cancer code in patient’s primary care record in the CPRD dataset (S1 Table) pertaining to the cancer diagnosis was assigned to be the date of diagnosis [12, 25] and the clinical record for the 12 month period preceding this date was studied. The ‘diagnostic interval’ was defined as the duration from the first occurrence of a symptom code in CPRD pertaining to a possible cancer to the date of cancer diagnosis, and was censored at 365 days. Hence, the diagnostic interval was calculated only for patients with identifiable symptom codes. The patients who were screen- or incidentally-detected, or who had emergency admissions without any symptom information were excluded. Although there have been reports of patients experiencing symptoms for more than a year before diagnosis [29], it is difficult to know whether very early symptoms genuinely arise from the cancer in question, or from benign or incidental conditions. We chose 365 days as a reasonable compromise in the absence of any methodological precedence [12] and it is in keeping with recently published consensus recommendations [13].

### Data analysis

We examined the relationships between diagnostic interval and each of age, gender and *NICE* status. Separate analyses were carried out for each cancer site as well as a single overall analysis that included all cancers.

Numbers and percentages of symptomatic patients in the dataset, males and females, and patients with either *NICE* or *non-NICE* symptoms [12] among the symptomatic patients for each cancer site are reported. The mean age at diagnosis is reported for each cancer site. The distribution of diagnostic interval was summarised, reporting the mean, standard deviation, median, inter-quartile range (IQR), and 90^th^ centile. Median, IQR and 90^th^ centiles are shown as the preferred method for describing these skewed data, but comparisons across sub-groups are based on mean diagnostic interval (using linear regression models with diagnostic interval as the outcome and age, gender, and *NICE* status as predictors) as this was the parameter we wanted to make inferences for. Because the diagnostic interval distributions were skewed, we validated the linear regression results by constructing bias-corrected accelerated bootstrap confidence intervals for the mean differences (regression coefficients) as these are robust to non-normality [30]. As the bootstrap confidence intervals were virtually the same as the regression model-based confidence intervals we report results from the latter analysis. The four gender-specific cancers (breast, cervical, endometrial and testicular) were omitted from the analyses of diagnostic intervals against gender.

Unadjusted (crude) linear regression models were fitted in which only one predictor was included and multivariable models in which all three of age, gender, and *NICE* status were included as predictors. We focus on the multivariable analyses as primary. Fractional polynomial models were used to check that the continuous predictor, age, had a linear relationship with diagnostic interval. Where the relationship was linear we reported the increase in mean diagnostic interval for every 10 year increase in age. Where the relationship was non-linear we divided the patient sample into five equal sized age categories based on the quintiles and used age as a categorical predictor in the linear regression model, comparing the mean of each of the four older categories to the youngest age category (reference category).

Where there was evidence at the 5% level of an association between diagnostic interval and the age and gender predictors, tests of interaction were undertaken to explore whether the relationships differ between categories defined by *NICE* status. All data manipulation and analyses were performed using Stata 11.0 software (StataCorp. 2009. *Stata Statistical Software*: *Release 11*. College Station, TX: StataCorp LP.).

## Results

### Demographic and symptom profile of the study sample

33,008 patients had a new diagnosis of cancer during the study period; of these 18,618 (56.4%) had a recorded symptom in the 12 months before diagnosis, so were included in the analyses. Mean age varied among cancers ranging from 50.8 (SD 10.5) years for testicular to 73.5 (SD 10.4) years for bladder. Because the dataset only contained patients aged 40 years or more, the mean ages for those cancers also affecting younger people are artefactually high. Percentages of patients with symptoms varied among cancer sites with leukaemia having the lowest (19.5%) and oesophageal having the highest (75.4%) percentage of symptomatic patients respectively. Patients presenting with *NICE* symptoms were considerably more common than with *non-NICE* symptoms for all cancers except cervical. More males than females had symptoms for all the gender non-specific cancers except lymphoma (49.9%) and pancreatic (46.6%). The general characteristics of patients with no symptoms were similar to the symptomatic population in all cancers, though these data are not presented here as the focus of this study was symptomatic cancer patients. Table 1 summarises the patient demographic characteristics regarding age, gender and percentage of symptomatic patients in each cancer group in the dataset.

**Table 1:** Demographic characteristics of the patient population in the dataset.

Cancer site	Number diagnosed (n)	n (%)* with symptoms	n (%)** with *NICE* symptoms	Age at diagnosis			n (%)*** females
				Median	Mean	SD	
**Bladder**	2210	1519 (68.7)	1299 (85.5)	74	73.5	10.4	395 (26.0)
**Breast**	3147	1620 (51.5)	1316 (81.2)	66	67.1	13.3	1620 (100)
**Cervix**	421	150 (35.6)	68 (45.3)	57	59.4	14.9	150 (100)
**Colorectal**	6557	4363 (66.5)	2508 (57.5)	73	72	11.2	1984 (45.6)
**Gastric**	2021	1118 (55.3)	1002 (89.6)	74	72.9	10.8	397 (35.5)
**Head and Neck**	612	328 (53.6)	282 (86.0)	69	67.9	12.3	68 (20.7)
**Kidney**	1467	503 (34.3)	272 (54.0)	70	68.7	11.4	172 (34.2)
**Leukaemia**	1961	383 (19.5)	370 (96.6)	72	70.7	11.9	185 (48.3)
**Lung**	6552	4253 (64.9)	3816 (89.7)	73	72.1	10.3	1821 (42.8)
**Lymphoma**	2232	685 (30.7)	652 (95.2)	70	68.8	12	343 (50.1)
**Myeloma**	1158	500 (43.2)	497 (99.4)	72	71.4	10.8	218 (43.6)
**Oesophageal**	1842	1389 (75.4)	1314 (94.6)	72	71.0	11.1	464 (33.4)
**Pancreatic**	1370	946 (69.1)	859 (90.8)	73	71.9	11.7	505 (53.4)
**Testicular**	161	104 (64.6)	68 (65.4)	47	50.8	10.5	104 (100)
**Endometrial**	1297	757 (58.3)	571 (75.4)	66	66.9	10.9	757 (100)
**Pooled**	33008	18618 (56.4)	14894 (80.0)	72	70.6	11.7	6552 (41.0)

* Number of with symptoms as a fraction of the number with cancer diagnosis

**Number with *NICE* symptoms as a fraction of the number with symptoms (fraction of those analysed)

*** Number of females as a fraction of the number with symptoms (fraction of those analysed).

### Diagnostic interval distributions

Mean (SD) diagnostic intervals were shortest for testicular cancer (54.5 days (50.8)) and longest for myeloma (161.8 days (114.0)). The cancers with the shortest median diagnostic intervals (Table 2) were breast (27 days), testicular (41 days), and pancreatic (59 days); and those with the longest were myeloma (149 days), lung (113 days), and leukaemia (102 days). Similarly, the cancers with the shortest 90^th^ centile diagnostic intervals were testicular (113 days), breast (210 days), and cervical (228 days); and those with the longest were myeloma (334 days), lung (326 days), leukaemia (311 days) and gastric (310 days).

**Table 2:** Regression analysis of diagnostic intervals against age* in 15 cancers.

Cancer site	n	Diagnostic interval							
		Mean	SD	Median	Interquartile range	90th centile	Incremental change in days per 10 years of age		
							Mean (95% CI)		Adjusted* P value
							Crude	Adjusted*	
**Bladder**	1519	119.0	102.6	80	40 to 179	293	12.0	10.3 (5.5 to 15.1)	<0.001
**Breast**	1620	63.3	86.3	27	15 to 62	210	0.1	-1.3 (-3.8 to 1.2)	0.30
**Cervix**	150	98.8	89.6	67	30 to 145	228	-1.6	3.7 (-5.9 to 13.4)	0.44
**Colorectal**	4363	120.3	103.3	80	37 to 188	296	NA**	NA**	NA**
**Gastric**	1118	125.0	107.8	84	35 to 199	310	-5.0	-5.9 (-11.7 to -0.2)	0.04
**Head and Neck**	328	121.3	94.4	87	51 to 177	281	-2.9	-3.3 (-11.8 to 5.1)	0.43
**Kidney**	503	119.0	99.8	84	42 to 175	293	12.5	11.0 (3.4 to 18.6)	0.004
**Leukaemia**	383	133.5	113.9	102	28 to 230	311	17.8	18.5 (8.8 to 28.1)	<0.001
**Lung**	4253	147.4	113.5	113	45 to 249	326	10.2	10.1 (6.7 to 13.4)	<0.001
**Lymphoma**	685	130.0	103.3	99	44 to 209	298	2.5	2.8 (-3.5 to 9.0)	0.39
**Myeloma**	500	161.8	114.0	149	54 to 263	334	5.8	5.6 (-3.3 to 14.6)	0.21
**Oesophageal**	1389	125.6	108.2	83	35 to 207	308	-2.5	-3.0 (-8.1 to 2.1)	0.26
**Pancreatic**	946	96.5	93.5	59	26 to 145	248	-5.2	-6.0 (-11.2 to -0.7)	0.03
**Testicular**	104	54.5	50.8	41	20 to 66	113	4.5	2.7 (-6.2 to 11.5)	0.55
**Endometrial**	757	100.0	86.5	67	36 to 138	239	-2.4	0.4 (-5.4 to 6.1)	0.90
**Pooled^**	18618	121.2	106.6	79	35 to 195	302	7.9	7.8 (6.4 to 9.1)	<0.001

*Model adjusted for gender (where relevant) and *NICE* status

**Table 2b for analysis for colorectal site using age as a categorical predictor

^^^15987 for gender analysis in Table 3.

**Table 3:** Differences in diagnostic intervals across age categories* in colorectal cancer patients.

Age group categories	Diagnostic interval			
	Mean (SD)	Mean difference (95% CI) from youngest age category *		Adjusted** P value
		Crude	Adjusted**	
**40 to 62 (youngest)**	105.4 (95.3)	Reference	Reference	<0.001
**63 to 70**	109.3 (98.3)	3.8	4.3 (-5.3 to 13.9)	
**71 to 76**	118.4 (103.2)	13.0	14.0 (4.5 to 23.5)	
**77 to 81**	125.7 (104.6)	20.0	21.4 (11.9 to 31.0)	
**82+ (oldest)**	142.6 (103.3)	37.0	37.0 (27.4 to 46.6)	

*Youngest age category (40–62) was used as the reference for comparison

**Model adjusted for gender and *NICE* status.

### Diagnostic intervals and age

**Fig 1:**
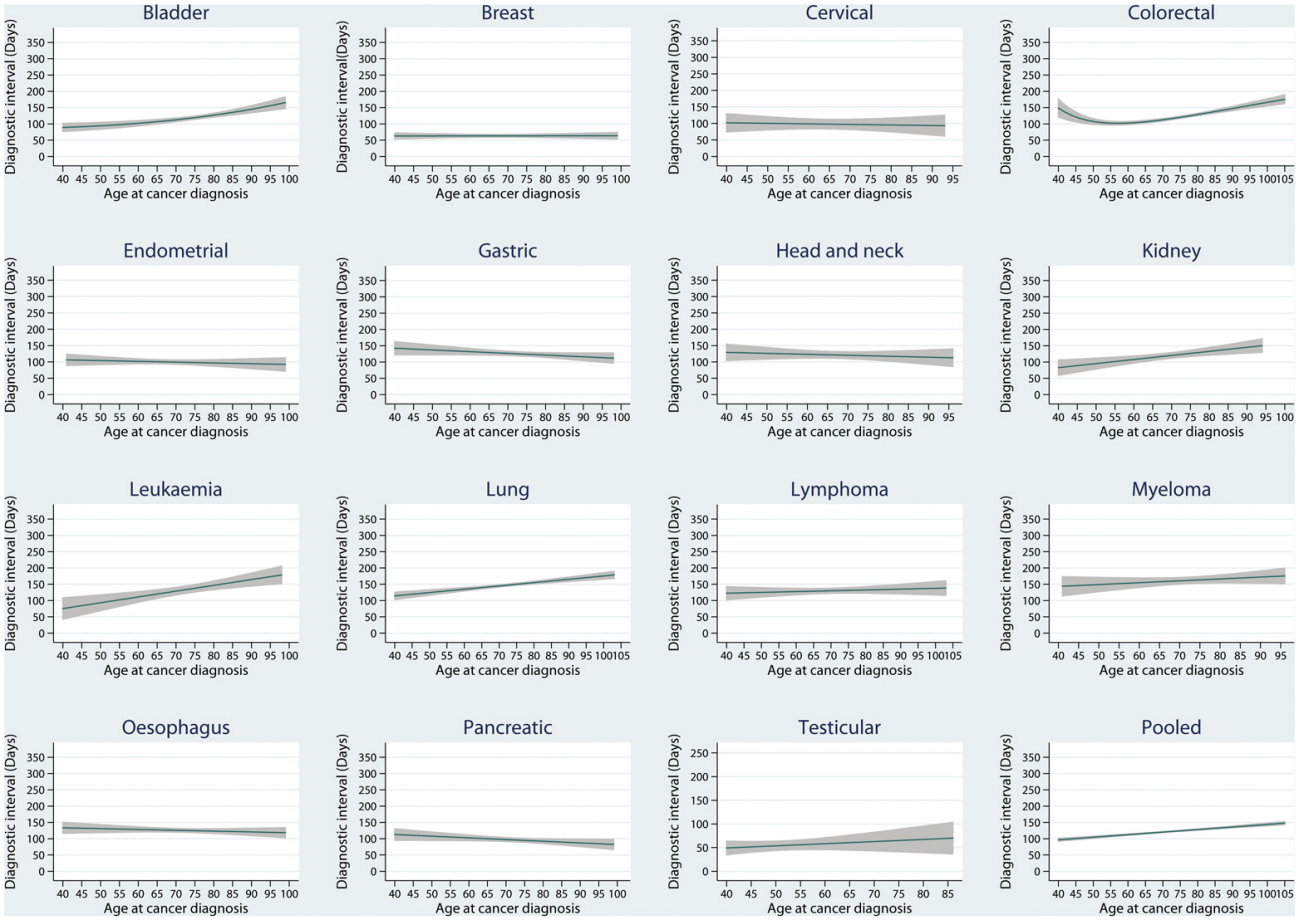
Line graphs to illustrate fitted relationships between diagnostic interval and age.

The results from fitted fractional polynomial linear regression models indicated that the relationship between age and diagnostic interval was linear for all cancers except colorectal (Fig 1), in which the age was hence analysed as a categorical predictor in the linear regression model.

The adjusted mean change in diagnostic interval per 10 year increase in age ranged from a 19 day increase for leukaemia to a 6 day decrease for pancreatic and gastric cancers (Table 2). There was evidence of a relationship between diagnostic interval and age for seven of the cancers in the multivariable analysis showing longer diagnostic interval with increasing age for five cancers (mean change per 10 year increase in age; 95% confidence interval; p value): bladder (10.3 days; 95% CI: 5.5 to 15.1; P<0.001), kidney (11.0 days; 95% CI: 3.4 to 18.6; P = 0.004), leukaemia (18.5 days; 95% CI: 8.8 to 28.1; P<0.001), lung (10.1 days; 95% CI: 6.7 to 13.4; P<0.001) and colorectal (P <0.001—see Table 3); whereas mean diagnostic interval was shorter for two of the cancers: gastric (-5.9 days; 95% CI: -11.7 to -0.2; P = 0.04) and pancreatic (-6.0 days; 95% CI: -11.2 to -0.7; P = 0.03). There were no significant differences in other cancers. Pooling the patients from all cancers (Table 2) resulted in strong evidence of a relationship showing longer diagnostic interval with increasing age (7.8 days per 10 year increase; 6.4 to 9.1; P<0.001). No evidence at the 5% level of significance was found of an interaction between age and *NICE* status for any cancer type.

### Diagnostic intervals and gender

The 11 gender non-specific cancers included 15,987 symptomatic patients (Table 4). There was evidence at the 5% level of significance of gender differences for six cancers, all showing longer diagnostic intervals for females: bladder (mean difference = 12.2 days; 0.8 to 23.6; P = 0.04), colorectal (10.4 days; 4.3 to 16.5; P = 0.001), gastric (14.3 days; 1.1 to 27.6; P = 0.03), head and neck (31.3 days; 6.2 to 56.5; P = 0.02), lung (8.0 days; 1.2 to 14.9; P = 0.02), and lymphoma (19.2 days; 3.8 to 34.7; P = 0.01). Pooling (Table 4) the patients from all cancers resulted in a gender difference overall, with longer mean diagnostic interval for females (8.9 days; 5.5 to 12.2; P<0.001). No evidence at the 5% level of significance was found of an interaction between gender and *NICE* status for any cancer type.

**Table 4:** Regression analysis of diagnostic interval by gender* in 11 gender non-specific cancers^^^.

Cancer site	Gender	n (%)	Diagnostic interval							
			Mean	SD	Median	Interquartile range	90th centile	Mean difference (95% CI)		Adjusted* P value
								Crude	Adjusted*	
**Bladder**	***Males***	1124 (74.0)	115.3	99.9	78	40 to 173	286	14.3	12.2 (0.8 to 23.6)	0.04
	***Females***	395 (26.0)	129.6	109.4	88	43 to 196	312			
**Colorectal**	***Males***	2379 (54.5)	114.1	100.8	74	35 to 173	287	13.7	10.4 (4.3 to 16.5)	0.001
	***Females***	1984 (45.6)	127.8	105.9	89	41 to 204	303			
**Gastric**	***Males***	721 (64.5)	119.9	106.0	77	35 to 182	306	14.3	14.3 (1.1 to 27.6)	0.03
	***Females***	397 (35.5)	134.1	110.7	98	37 to 216	320			
**Head and Neck**	***Males***	260 (79.3)	114.5	91.2	84	48 to 158	270	33.2	31.3 (6.2 to 56.5)	0.02
	***Females***	68 (20.7)	147.6	101.9	125	62 to 231	300			
**Kidney**	***Males***	331 (65.8)	113.8	98.2	78	38 to 162	284	15.2	8.1 (-10.4 to 26.6)	0.39
	***Females***	172 (34.2)	129.0	102.4	101	44 to 196	300			
**Leukaemia**	***Males***	198 (51.7)	133.0	112.2	101	31 to 226	309	1.1	-1.1 (-23.7 to 21.5)	0.92
	***Females***	185 (48.3)	134.1	115.9	104	27 to 237	312			
**Lung**	***Males***	2432 (57.2)	144.1	113.2	106	44 to 241	325	7.7	8.0 (1.2 to 14.9)	0.02
	***Females***	1821 (42.8)	151.8	113.9	122	48 to 255	327			
**Lymphoma**	***Males***	342 (49.9)	120.1	96.7	86	40 to 183	269	19.7	19.2 (3.8 to 34.7)	0.01
	***Females***	343 (50.1)	139.9	108.9	107	47 to 235	314			
**Myeloma**	***Males***	282 (56.4)	158.0	114.5	143	51 to 259	328	8.6	7.4 (-12.9 to 27.7)	0.47
	***Females***	218 (43.6)	166.6	113.4	155	63 to 273	338			
**Oesophagus**	***Males***	925 (66.6)	123.7	107.4	80	35 to 197	308	5.5	6.2 (-5.9 to 18.3)	0.32
	***Females***	464 (33.4)	129.3	109.6	90	35 to 221	307			
**Pancreatic**	***Males***	441 (46.6)	98.9	95.9	63	25 to 150	254	-4.4	-4.1 (-16.0 to 7.9)	0.50
	***Females***	505 (53.4)	94.5	91.4	56	27 to 139	241			
**Pooled** ^	***Males***	9435 (59.0)	124.6	106.0	84	38 to 199	305	10.2	8.9 (5.5 to 12.2)	<0.001
	***Females***	6552 (41.0)	134.8	109.6	97	42 to 223	313			

*Model adjusted for age and *NICE* status

**Number too small in *NICE* status to generate p value

^^^15987 for analysis of gender non-specific cancers.

### Diagnostic intervals and *NICE* status

All 15 cancers were included in these analyses (Table 5). There was evidence that the diagnostic interval was longer for *non-NICE* symptoms in 10 of the 15 cancers: bladder (mean difference = 62.9; 48.7 to 77.2; P<0.001), breast (115.1; 105.9 to 124.3; P<0.001), cervical (60.3; 31.6 to 89.0; P<0.001), colorectal (25.8; 19.6 to 31.9; P<0.001), gastric (24.1; 3.4 to 44.8; P = 0.02), kidney (22.1; 4.5 to 39.7; P = 0.01), oesophageal (67.0; 42.1 to 92.0; P<0.001), pancreatic (48.6; 28.1 to 69.1; P<0.001), testicular (36.7; 17.0 to 56.4; P< 0.001), and endometrial (73.8; 60.3 to 87.3; P<0.001). Pooling (Table 5) the patients from all cancers resulted in strong evidence of an overall increase in diagnostic interval for *non-NICE* symptoms (27.7 days; 23.9 to 31.5; P<0.001).

**Table 5:** Regression analysis of diagnostic intervals by *NICE* status in 15 cancers.

Cancer site	*NICE* status	n (%)	Diagnostic interval							
			Mean	SD	Median	Interquartile range	90th centile	Mean difference (95% CI)		P value*
								Crude	Adjusted*	
**Bladder**	***NICE***	1299 (85.5)	109.5	99.0	71	37 to 155	284	62.9	62.9 (48.7 to 77.2)	<0.001
	***non-NICE***	220 (14.5)	175.0	105.9	172	87 to 268	327			
**Breast**	***NICE***	1316 (81.2)	41.8	59.7	22	14 to 42	83	114.9	115.1 (105.9 to 124.3)	<0.001
	***non-NICE***	304 (18.8)	156.7	116.7	138	54 to 263	337			
**Cervical**	***NICE***	68 (45.3)	66.6	63.2	43	27 to 90	179	57.4	60.3 (31.6 to 89.0)	<0.001
	***non-NICE***	82 (54.7)	124.0	99.8	90	49 to 192	273			
**Colorectal**	***NICE***	2508 (57.5)	109.4	98.2	69	35 to 163	279	25.7	25.8 (19.6 to 31.9)	<0.001
	***non-NICE***	1855 (42.5)	135.1	108.2	100	43 to 218	310			
**Gastric**	***NICE***	1002 (89.6)	122.4	106.9	80	35 to 195	309	25.0	24.1 (3.4 to 44.8)	0.02
	***non-NICE***	116 (10.4)	147.3	113.5	117	48 to 244	329			
**Head and Neck**	***NICE***	282 (86.0)	117.8	91.9	84	50 to 167	272	25.5	22.6 (-6.8 to 52.0)	0.13
	***non-NICE***	46 (14.0)	143.3	106.8	101	55 to 218	312			
**Kidney**	***NICE***	272 (54.0)	106.9	94.0	76	39 to 145	281	26.2	22.1 (4.5 to 39.7)	0.01
	***non-NICE***	231 (46.0)	133.2	104.7	104	44 to 211	296			
**Leukaemia**	***NICE***	370 (96.6)	134.4	114.3	103	28 to 231	312	-26.4	-39.5 (-102.2 to 23.1)	0.21
	***non-NICE***	13 (3.4)	108.0	103.8	57	28 to 166	262			
**Lung**	***NICE***	3816 (89.7)	146.2	113.8	112	44 to 248	327	11.3	8.8 (-2.4 to 20.0)	0.12
	***non-NICE***	437 (10.3)	157.5	110.5	139	60 to 257	323			
**Lymphoma**	***NICE***	652 (95.2)	131.1	102.9	100	46 to 210	295	-23.0	-22.8 (-58.4 to 14.0)	0.23
	***non-NICE***	33 (4.8)	108.1	111.0	44	24 to 157	310			
**Myeloma**	***NICE***	497 (99.4)	162.2	113.9	149	54 to 263	334	-68.8	-65.8 (-195.8 to 64.2)	0.32
	***non-NICE***	3 (0.6)	93.3	130.9	28	8 to 244	244			
**Oesophagus**	***NICE***	1314 (94.6)	121.9	106.5	79	34 to 200	300	67.2	67.0 (42.1 to 92.0)	<0.001
	***non-NICE***	75 (5.4)	189.1	117.0	169	89 to 306	352			
**Pancreatic**	***NICE***	859 (90.8)	92.3	91.4	56	25 to 131	242	67.2	48.6 (28.1 to 69.1)	<0.001
	***non-NICE***	87 (9.2)	138.6	104.0	115	43 to 211	293			
**Testicular**	***NICE***	68 (65.4)	41.5	32.5	35	20 to 57	77	37.4	36.7 (17.0 to 56.4)	<0.001
	***non-NICE***	36 (34.6)	78.9	68.0	56	29 to 110	182			
**Endometrial**	***NICE***	571 (75.4)	81.9	69.9	58	33 to 107	176	72.9	73.8 (60.3 to 87.3)	<0.001
	***non-NICE***	186 (24.6)	155.6	107.5	135	63 to 239	320			
**Pooled**	***NICE***	14894 (80.0)	115.7	105.1	73	32 to 183	296	27.5	27.7 (23.9 to 31.5)	<0.001
	***non-NICE***	3724 (20.0)	143.3	109.5	113	48 to 233	318			

*Model adjusted for age and gender (where relevant).

## Discussion

### Summary of the main findings

The overall findings were that longer diagnostic intervals are associated with increased age, female gender and *non-NICE* symptoms. Not all cancer sites had these associations: for older age, longer diagnostic intervals were observed in five cancers (bladder, colorectal, kidney, leukaemia and lung) but shorter diagnostic intervals in two cancers (gastric and pancreatic). Gender analyses showed females had longer diagnostic interval than males, with significant evidence at the 5% level in six cancers (bladder, colorectal, gastric, head and neck, lung, and lymphoma). Presentation of a *NICE* symptom before diagnosis was associated with shorter diagnostic intervals in 10 of the 15 cancers (bladder, breast, cervical, colorectal, gastric, kidney, oesophagus, pancreatic, testicular and endometrial). Data combined from all cancers included in this study and analysed together showed that the diagnostic interval was longer for older patients, females and *non-NICE* symptoms.

### Comparison with existing literature

This is the first study of this type to report the association between diagnostic interval and age and gender for patients with cancer. There was evidence that diagnostic interval increased with older age in five of 15 cancers. This finding is contradictory to a previous report [18] where longer diagnostic delays were reported for younger age groups, although this may be explained by methodological differences such as: their data were collected from patient surveys, whereas ours were GP-coded and collected from primary care consultations; they used different measures to analyse the data; there was a difference in the definition of diagnostic interval: number of days from first symptomatic presentation to date of diagnosis was used in our study, whereas ‘primary care delay’ was used in their study (derived by subtracting referral delay from the duration from noticing first symptoms to appointment by hospital doctor, based on patient recollection of these events). The findings of our study align with those of a recent report of a project piloted in five UK cancer network jurisdictions aimed, among others, at testing new methods of clinical assessment of older cancer patients. One of the main findings was that older cancer patients were being discriminated against, with care and treatment being determined based on age and not needs [21]. Other potential reasons to explain these findings include: changes in the nature, perception and presentation of symptoms with age [31], although this has not been shown in previous studies [32]; increasing age-related co-morbidity with concurrent treatment(s) masking potential cancer symptoms [14, 31]; varying tumour biology and aggressiveness with age [33] and/or gender [34]; a reluctance by GPs to refer or investigate older and frailer people [35–37]; and differing age specific patterns in willingness to be referred for onward investigation by the patients [38].

Longer diagnostic interval and advanced stage at diagnosis in females have been reported before for some cancers [18, 19, 39] and our findings are in keeping with these; this is a useful corroboration as our data source is different. The significant relationship of female gender with longer diagnostic intervals in six of the 11 gender non-specific cancers analysed in our study supports the findings of disparities reported in other studies that females might delay seeking help when they detect or realise the presence of potential cancer related symptoms [14, 19] as well as other chronic conditions such as heart disease [40], COPD [41] and others [42]. Although, this trend appears to be improving [43], it still highlights the need for a deeper understanding of this multi-dimensional phenomenon [44] of gender difference to tailor interventions according to patients’ socioeconomic and cultural background [45], especially when females are reported to be keener on seeking more health related information [46], and appear to be more receptive [47]. This finding also highlights the fact that symptoms should not be overlooked by the health care professionals based on patients’ gender only.

This study adds to previous findings that ‘alarm’ symptoms that qualify a patient for urgent referral (*NICE*) had shorter diagnostic interval than the ‘vague’ symptoms (*non-NICE*) [9, 12] indicating that the symptoms that were already getting a good service are getting an even better one [31], and their prioritisation over more vague symptoms may lead to a ‘slow track’ for diagnosis [12, 48, 49].

### Strengths and limitations

In the UK, over 95% of the population is uniquely registered with only one general practice. Hence, the population data derived from the GP system is highly representative of the general population. We used a large, longitudinal UK general practice dataset, which has previously been used for cancer diagnostic studies [23, 24] and has been validated for diagnostic coding accuracy of upto 95% in recent systematic reviews [27, 50].

Though our definition of diagnostic interval aligns with recent recommendations on the design and conduct of studies using such datasets [13], there are methodological weaknesses in measuring diagnostic interval from electronic records [12]. This study used CPRD codes to extract symptom and cancer diagnosis dates from the dataset. The cancer diagnostic codes are usually entered in the GP system by the practice staff upon receipt of the diagnostic confirmation letter from a hospital bearing the date of diagnosis. There is a possibility at this stage that the date of the letter itself or the date of coding entry might erroneously be entered as the date of cancer diagnosis. This may affect the diagnostic interval in some cases. Likewise, some cancer diagnoses will have been unrecorded or recorded incorrectly, leading to either such cases being excluded from our analysis or might have affected the correct diagnostic interval calculations respectively. These effects, though, are unlikely to affect a large proportion of the study population when the CPRD databases have been validated to show a diagnostic coding accuracy of upto 95% recently [27, 50]. Similarly some symptoms might not have been recorded, or recorded in a less accessible field (so-called ‘free-text’), although this may not be important because a recent CPRD study indicated that free-text data usually only confirms what is entered in an accessible coded form [51], and electronic records have been found to be of similar quality to paper records [52]. Furthermore, some cancers might have presented with different or atypical symptoms not included in our defined list. Also, we assumed that all the symptoms in our list represented the symptomatic presentation of the cancer; however some may have been co-incidental.

Although we were unable to specifically identify screen-detected patients, most would have had no symptoms, and would therefore have been correctly excluded. Low proportions of symptomatic patients in some cancers, such as breast, can be explained by the fact that between 39–46% patients can be screen-detected as reported in other UK studies using different data sources [53, 54]; others could present with atypical symptoms or as emergency admissions.

The cut-off point for symptoms at 12 months prior to the date of diagnosis was based on the judgement that very few would have had a diagnostic interval longer than this. If we had extended the time cut-off we would have picked more patients whose symptoms might not have been related to subsequent cancer diagnosis, but we equally would have captured more patients with genuine diagnostic interval of greater than one year. There may also be variation between cancers; however, for consistency and in the absence of any methodological precedents, we used the time period of 12 months for all the cancers as a compromise.

Patients under the age of 40 were not included. This was based on a practical decision because of the rarity of cancer diagnoses in this group; only 10% of all new cases in the UK occur in the age group 25–49 [55]; and if they do occur, may be atypical or part of a familial syndrome [56, 57]. This approach is in keeping with similar primary care studies [12, 25]. Apart from this, the age and symptom profile as well as male to female ratios in our datasets are similar to other national cancer surveillance systems [55, 58] indicating that the sample was representative of the UK cancer population.

The authors would urge caution in interpreting and generalising the findings of this study keeping in mind the inherent methodological limitations of analysing retrospective electronic data such as completeness, accuracy etc. We would also reiterate that our results would only apply to cases that had symptomatic presentation before the date of diagnosis, hence some patients with emergency admissions who had missing symptom information in their records would have been excluded, though they might have had shorter diagnostic intervals. Similarly, screen- or incidentally-detected cancer patients would have been excluded as well. These artefacts would limit the generalisability of the findings. We also acknowledge that clinical heterogeneity within certain cancer groups in our study (e.g. leukaemia, head and neck), may also limit the generalisability of our findings.

### Implications

Interventions aimed at reducing cancer diagnostic intervals should be tailored to address inequalities in certain age and/or gender groups. This study has identified specific cancer sites where such action would be of benefit. We have also provided a baseline against which future intervention effects as well as evaluation outcomes can be assessed. More work is needed to understand the complex interaction between age, gender and types of symptoms and diagnostic intervals, their effects on stage at diagnosis, and the types of interventions needed to address the inequalities.

## Conclusions

Diagnostic interval has been shown to vary with age, gender and *NICE* status across 15 different cancers. For some, there appear to be little age and/or gender differences. However, increasing age for bladder, colorectal, kidney, leukaemia, and lung cancers; female gender for bladder, colorectal, gastric, head and neck, lung, and lymphoma cancers; and *non-NICE* symptoms for 10 of the 15 cancers analysed in this study were associated with longer diagnostic intervals.

## Supporting Information

S1 Table Cancer diagnosis Read Codes. (DOCX)

S2 Table Cancer site specific symptom Read Codes. (DOCX)

S3 Table Cancer site non-specific symptom Read Codes. (DOCX)

S4 Table List of symptoms by cancer site. (DOCX)

S5 Table List of *NICE* symptom categories by cancer site. (DOCX)
