# Supplementary material for: Age and Gender Variations in Cancer Diagnostic Intervals in 15 Cancers: Analysis of Data from the UK Clinical Practice Research Datalink
Source: PLoS One. 2015 May 15;10(5):e0127717. doi: 10.1371/journal.pone.0127717 (PMC4433335; doi:10.1371/journal.pone.0127717)
Supplement: S1 Table — (DOCX) [file pone.0127717.s001.docx]

**S1 Table. Cancer diagnosis Read Codes**

| **Read code** | **Read term** |
| --- | --- |
| **Colorectal** |  |
| B140.00 | Malignant neoplasm of rectosigmoid junction |
| B13z.00 | Malignant neoplasm of colon nos |
| B804100 | Carcinoma in situ of rectum |
| B18y200 | Malignant neoplasm of mesorectum |
| B803.00 | Carcinoma in situ of colon |
| B575z00 | Secondary malig neop of large intestine or rectum nos |
| B803700 | Carcinoma in situ of splenic flexure of colon |
| B14y.00 | Malig neop other site rectum, rectosigmoid junction and anus |
| B804.00 | Carcinoma in situ of rectum and rectosigmoid junction |
| B803300 | Carcinoma in situ of sigmoid colon |
| B134.00 | Malignant neoplasm of caecum |
| B141.00 | Malignant neoplasm of rectum |
| B804000 | Carcinoma in situ of rectosigmoid junction |
| B141.12 | Rectal carcinoma |
| B575.00 | Secondary malignant neoplasm of large intestine and rectum |
| B803z00 | Carcinoma in situ of colon nos |
| B803100 | Carcinoma in situ of transverse colon |
| B803200 | Carcinoma in situ of descending colon |
| B130.00 | Malignant neoplasm of hepatic flexure of colon |
| B133.00 | Malignant neoplasm of sigmoid colon |
| B138.00 | Malignant neoplasm, overlapping lesion of colon |
| B18y000 | Malignant neoplasm of mesocolon |
| B13y.00 | Malignant neoplasm of other specified sites of colon |
| B136.00 | Malignant neoplasm of ascending colon |
| B575100 | Secondary malignant neoplasm of rectum |
| B13..00 | Malignant neoplasm of colon |
| B141.11 | Carcinoma of rectum |
| B132.00 | Malignant neoplasm of descending colon |
| B1z0.11 | Cancer of bowel |
| B13z.11 | Colonic cancer |
| B803400 | Carcinoma in situ of caecum |
| B803600 | Carcinoma in situ of ascending colon |
| B804z00 | Carcinoma in situ of rectum or rectosigmoid junction nos |
| B134.11 | Carcinoma of caecum |
| B575000 | Secondary malignant neoplasm of colon |
| B14z.00 | Malignant neoplasm rectum,rectosigmoid junction and anus nos |
| B803000 | Carcinoma in situ of hepatic flexure of colon |
| B137.00 | Malignant neoplasm of splenic flexure of colon |
| B131.00 | Malignant neoplasm of transverse colon |
| B14..00 | Malignant neoplasm of rectum, rectosigmoid junction and anus |
|  |  |
| **Lung** |  |
| B2zy.00 | Malignant neoplasm of other site of respiratory tract |
| Bbp5.00 | [m]epithelioid mesothelioma, malignant |
| B812300 | Carcinoma in situ of middle lobe bronchus and lung |
| B26..00 | Malignant neoplasm, overlap lesion of resp & intrathor orgs |
| Bbp7.00 | [m]mesothelioma, biphasic type, malignant |
| B812400 | Carcinoma in situ of lower lobe bronchus and lung |
| Bb5s212 | [m]bronchiolar carcinoma |
| B570.00 | Secondary malignant neoplasm of lung |
| Bbpx.00 | [m]mesothelioma, unspecified |
| B561700 | Secondary and unspec malig neop inferior tracheobronchial ln |
| Bbp1.00 | [m]mesothelioma, malignant |
| B2...00 | Malig neop of respiratory tract and intrathoracic organs |
| B223100 | Malignant neoplasm of middle lobe of lung |
| B561800 | Secondary and unspec malig neop bronchopulmonary lymph nodes |
| B221000 | Malignant neoplasm of carina of bronchus |
| B22y.00 | Malignant neoplasm of other sites of bronchus or lung |
| B222z00 | Malignant neoplasm of upper lobe, bronchus or lung nos |
| B232.00 | Mesothelioma of pleura |
| Byu5100 | [x]mesothelioma, unspecified |
| B812100 | Carcinoma in situ of main bronchus |
| B551z00 | Malignant neoplasm of thorax nos |
| B812z00 | Carcinoma in situ of bronchus or lung nos |
| B222100 | Malignant neoplasm of upper lobe of lung |
| B22z.00 | Malignant neoplasm of bronchus or lung nos |
| B222.00 | Malignant neoplasm of upper lobe, bronchus or lung |
| B551.00 | Malignant neoplasm of thorax |
| B223.00 | Malignant neoplasm of middle lobe, bronchus or lung |
| Bblm.00 | [m]pulmonary blastoma |
| B221.00 | Malignant neoplasm of main bronchus |
| B561600 | Secondary and unspec malig neop superfic tracheobronchial ln |
| L1970p | Metastasis pulmonary |
| B224100 | Malignant neoplasm of lower lobe of lung |
| Bbtl.00 | [m]intravascular bronchial alveolar tumour |
| B222.11 | Pancoast's syndrome |
| B23..00 | Malignant neoplasm of pleura |
| Bbp9.00 | [m]cystic mesothelioma |
| B226.00 | Mesothelioma |
| Byu5011 | [x]mesothelioma of lung |
| B812200 | Carcinoma in situ of upper lobe bronchus and lung |
| B225.00 | Malignant neoplasm of overlapping lesion of bronchus & lung |
| Bb1k.00 | [m]oat cell carcinoma |
| Bb5s200 | [m]bronchiolo-alveolar adenocarcinoma |
| B221z00 | Malignant neoplasm of main bronchus nos |
| B22z.11 | Lung cancer |
| B812000 | Carcinoma in situ of carina of bronchus |
| B812.00 | Carcinoma in situ of bronchus and lung |
| B222000 | Malignant neoplasm of upper lobe bronchus |
| B223000 | Malignant neoplasm of middle lobe bronchus |
| B224000 | Malignant neoplasm of lower lobe bronchus |
| B224.00 | Malignant neoplasm of lower lobe, bronchus or lung |
| B22..00 | Malignant neoplasm of trachea, bronchus and lung |
| B224z00 | Malignant neoplasm of lower lobe, bronchus or lung nos |
| Bbp3.00 | [m]fibrous mesothelioma, malignant |
| B223z00 | Malignant neoplasm of middle lobe, bronchus or lung nos |
| Byu2000 | [x]malignant neoplasm of bronchus or lung, unspecified |
| B221100 | Malignant neoplasm of hilus of lung |
|  |  |
| **Breast** |  |
| Bb91.00 | [m]infiltrating duct carcinoma |
| B34..00 | Malignant neoplasm of female breast |
| B34z.00 | Malignant neoplasm of female breast nos |
| B3...00 | Malig neop of bone, connective tissue, skin and breast |
| Bb94.11 | [m]secretory breast carcinoma |
| B3z..00 | Malig neop of bone, connective tissue, skin and breast nos |
| Bb85111 | [m]krukenberg tumour |
| B34y.00 | Malignant neoplasm of other site of female breast |
| B544.00 | Malignant neoplasm of carotid body |
| Bb91100 | [m]infiltrating duct and lobular carcinoma |
| B34yz00 | Malignant neoplasm of other site of female breast nos |
| B830000 | Lobular carcinoma in situ of breast |
| B35z000 | Malignant neoplasm of ectopic site of male breast |
| Bb9j.00 | [m]paget's disease, mammary |
| B35zz00 | Malignant neoplasm of male breast nos |
| B342.00 | Malignant neoplasm of upper-inner quadrant of female breast |
| B335200 | Malignant neoplasm of skin of breast |
| B350000 | Malignant neoplasm of nipple of male breast |
| Bb9k000 | [m]paget's disease and intraductal carcinoma of breast |
| Bb94.00 | [m]juvenile breast carcinoma |
| B830.00 | Carcinoma in situ of breast |
| Bb96.00 | [m]noninfiltrating intraductal papillary adenocarcinoma |
| B343.00 | Malignant neoplasm of lower-inner quadrant of female breast |
| B83..00 | Carcinoma in situ of breast and genitourinary system |
| B3...11 | Carcinoma of bone, connective tissue, skin and breast |
| B35..00 | Malignant neoplasm of male breast |
| Bb9f.00 | [m]lobular carcinoma nos |
| Bb9e000 | [m]intraductal carcinoma and lobular carcinoma in situ |
| B34y000 | Malignant neoplasm of ectopic site of female breast |
| B325100 | Malignant melanoma of breast |
| Bb9k.00 | [m]paget's disease and infiltrating breast duct carcinoma |
| B825000 | Carcinoma in situ of skin of breast |
| B341.00 | Malignant neoplasm of central part of female breast |
| B350.00 | Malignant neoplasm of nipple and areola of male breast |
| Ba03.00 | Neoplasm of unspecified nature of breast |
| B582600 | Secondary malignant neoplasm of skin of breast |
| B345.00 | Malignant neoplasm of lower-outer quadrant of female breast |
| B830100 | Intraductal carcinoma in situ of breast |
| Bb90.00 | [m]intraductal carcinoma, noninfiltrating nos |
| B58y000 | Secondary malignant neoplasm of breast |
| B340000 | Malignant neoplasm of nipple of female breast |
| B344.00 | Malignant neoplasm of upper-outer quadrant of female breast |
| B34..11 | Ca female breast |
| B335100 | Malignant neoplasm of skin of chest, excluding breast |
| B350100 | Malignant neoplasm of areola of male breast |
| B340z00 | Malignant neoplasm of nipple or areola of female breast nos |
| B3y..00 | Malig neop of bone, connective tissue, skin and breast os |
| Bb9j.11 | [m]paget's disease, breast |
| Byufg00 | [x]other carcinoma in situ of breast |
| B340100 | Malignant neoplasm of areola of female breast |
| B350z00 | Malignant neoplasm of nipple or areola of male breast nos |
| B35z.00 | Malignant neoplasm of other site of male breast |
| B340.00 | Malignant neoplasm of nipple and areola of female breast |
| B346.00 | Malignant neoplasm of axillary tail of female breast |
| B347.00 | Malignant neoplasm, overlapping lesion of breast |
| Byu6.00 | [x]malignant neoplasm of breast |
|  |  |
| **Pancreas** |  |
| Bb5b511 | [m]alpha-cell tumour,malignant |
| Bb5y300 | [m]apudoma |
| Bb5b200 | [m]insulinoma nos |
| B162.00 | Malignant neoplasm of ampulla of vater |
| B808700 | Carcinoma in situ of sphincter of oddi |
| Bb5b311 | [m]beta-cell tumour, malignant |
| Bb5cz00 | [m]gastrinoma or carcinoma nos |
| B161300 | Malignant neoplasm of sphincter of oddi |
| B174.00 | Malignant neoplasm of islets of langerhans |
| B17z.00 | Malignant neoplasm of pancreas nos |
| Bb81h00 | [m]papillary mucinous cystadenocarcinoma |
| Bb81800 | [m]papillary serous cystadenocarcinoma |
| Bb5b500 | [m]glucagonoma, malignant |
| Bb81200 | [m]serous cystadenocarcinoma, nos |
| Bb81b00 | [m]serous surface papillary carcinoma |
| B17..00 | Malignant neoplasm of pancreas |
| Bb5b011 | [m]nesidioblastoma |
| Bb5b600 | [m]mixed islet cell and exocrine adenocarcinoma |
| B717011 | Endocrine tumour of pancreas |
| B17y.00 | Malignant neoplasm of other specified sites of pancreas |
| B175.00 | Malignant neoplasm, overlapping lesion of pancreas |
| Bblk.00 | [m]pancreatoblastoma |
| B80z000 | Carcinoma in situ of pancreas |
| Bb5b300 | [m]insulinoma, malignant |
| Bb5b100 | [m]islet cell carcinoma |
| B173.00 | Malignant neoplasm of pancreatic duct |
| B172.00 | Malignant neoplasm of tail of pancreas |
| Bb5c000 | [m]gastrinoma nos |
| B171.00 | Malignant neoplasm of body of pancreas |
| B808600 | Carcinoma in situ of ampulla of vater |
| Bb81e11 | [m]pseudomucinous adenocarcinoma |
| Bb81e00 | [m]mucinous cystadenocarcinoma nos |
| Bb81500 | [m]papillary cystadenocarcinoma, nos |
| B170.00 | Malignant neoplasm of head of pancreas |
| B17y000 | Malignant neoplasm of ectopic pancreatic tissue |
| B17yz00 | Malignant neoplasm of specified site of pancreas nos |
| Bb5c100 | [m]gastrinoma, malignant |
| Bb5c.00 | [m]gastrinoma and carcinomas |
| Bb5b400 | [m]glucagonoma nos |
|  |  |
| **Oesophagus** |  |
| B103.00 | Malignant neoplasm of upper third of oesophagus |
| B110100 | Malignant neoplasm of cardio-oesophageal junction of stomach |
| B104.00 | Malignant neoplasm of middle third of oesophagus |
| B801200 | Carcinoma in situ of lower 1/3 oesophagus |
| B801100 | Carcinoma in situ of middle 1/3 oesophagus |
| B10y.00 | Malignant neoplasm of other specified part of oesophagus |
| B10z.00 | Malignant neoplasm of oesophagus nos |
| B801z00 | Carcinoma in situ of oesophagus nos |
| B801.00 | Carcinoma in situ of oesophagus |
| B10..00 | Malignant neoplasm of oesophagus |
| B102.00 | Malignant neoplasm of abdominal oesophagus |
| B100.00 | Malignant neoplasm of cervical oesophagus |
| B801000 | Carcinoma in situ of upper 1/3 oesophagus |
| B10z.11 | Oesophageal cancer |
| B105.00 | Malignant neoplasm of lower third of oesophagus |
| B110111 | Malignant neoplasm of gastro-oesophageal junction |
| B106.00 | Malignant neoplasm, overlapping lesion of oesophagus |
| B101.00 | Malignant neoplasm of thoracic oesophagus |
|  |  |
| **Stomach** |  |
| B802000 | Carcinoma in situ of cardia of stomach |
| B113.00 | Malignant neoplasm of fundus of stomach |
| B11z.00 | Malignant neoplasm of stomach nos |
| B110.00 | Malignant neoplasm of cardia of stomach |
| B111.00 | Malignant neoplasm of pylorus of stomach |
| B110000 | Malignant neoplasm of cardiac orifice of stomach |
| B11..11 | Gastric neoplasm |
| B113.00 | Malignant neoplasm of fundus of stomach |
| B111.00 | Malignant neoplasm of pylorus of stomach |
| B802.00 | Carcinoma in situ of stomach |
| B111100 | Malignant neoplasm of pyloric canal of stomach |
| B802100 | Carcinoma in situ of fundus of stomach |
| B802z00 | Carcinoma in situ of stomach nos |
| B110z00 | Malignant neoplasm of cardia of stomach nos |
| B116.00 | Malignant neoplasm of greater curve of stomach unspecified |
| B802200 | Carcinoma in situ of body of stomach |
| B11..00 | Malignant neoplasm of stomach |
| B111z00 | Malignant neoplasm of pylorus of stomach nos |
| B114.00 | Malignant neoplasm of body of stomach |
| Bb55.00 | [m]linitis plastica |
| B11yz00 | Malignant neoplasm of other specified site of stomach nos |
| B111000 | Malignant neoplasm of prepylorus of stomach |
| B11y100 | Malignant neoplasm of posterior wall of stomach nec |
| B112.00 | Malignant neoplasm of pyloric antrum of stomach |
| B115.00 | Malignant neoplasm of lesser curve of stomach unspecified |
| B117.00 | Malignant neoplasm, overlapping lesion of stomach |
| B11y.00 | Malignant neoplasm of other specified site of stomach |
| B11y000 | Malignant neoplasm of anterior wall of stomach nec |
| B110100 | Malignant neoplasm of cardio-oesophageal junction of stomach |
| B104.00 | Malignant neoplasm of middle third of oesophagus |
| B10..00 | Malignant neoplasm of oesophagus |
|  |  |
| **Endometrial** |  |
| B430000 | Malignant neoplasm of cornu of corpus uteri |
| B443.00 | Malignant neoplasm of parametrium |
| B430100 | Malignant neoplasm of fundus of corpus uteri |
| B44y.00 | Malignant neoplasm of other site of uterine adnexa |
| B430z00 | Malignant neoplasm of corpus uteri nos |
| Bbl0.00 | [m]endometrial stromal sarcoma |
| Bbk0700 | [m]myxoid leiomyosarcoma |
| B431000 | Malignant neoplasm of lower uterine segment |
| B832.11 | Carcinoma in situ of body of uterus |
| B43z.00 | Malignant neoplasm of body of uterus nos |
| B430.00 | Malignant neoplasm of corpus uteri, excluding isthmus |
| B43..00 | Malignant neoplasm of body of uterus |
| B833100 | Carcinoma in situ of fallopian tube |
| B432.00 | Malignant neoplasm of overlapping lesion of corpus uteri |
| B832.00 | Carcinoma in situ of other and unspecified parts of uterus |
| B58y100 | Secondary malignant neoplasm of uterus |
| Bbk0311 | [m]leiomyoblastoma |
| B40..00 | Malignant neoplasm of uterus, part unspecified |
| B431z00 | Malignant neoplasm of isthmus of uterine body nos |
| B43y.00 | Malignant neoplasm of other site of uterine body |
| B441.00 | Malignant neoplasm of fallopian tube |
| B832000 | Carcinoma in situ of endometrium |
| B430200 | Malignant neoplasm of endometrium of corpus uteri |
| B432.00 | Malignant neoplasm of overlapping lesion of corpus uteri |
| Bb5j200 | [m]endometrioid carcinoma |
| Bbk0200 | [m]leiomyosarcoma nos |
| B431.00 | Malignant neoplasm of isthmus of uterine body |
| B430300 | Malignant neoplasm of myometrium of corpus uteri |
| B430211 | Malignant neoplasm of endometrium |
| Byu7000 | [x]malignant neoplasm of uterine adnexa, unspecified |
| B44z.00 | Malignant neoplasm of uterine adnexa nos |
|  |  |
| **Cervical** |  |
| Bb2n.00 | [m]intraepit neop,grade iii,of cervix, vulva and vagina |
| B410100 | Malignant neoplasm of endocervical gland |
| B41z.00 | Malignant neoplasm of cervix uteri nos |
| B831100 | Carcinoma in situ of exocervix |
| B831.11 | Cin iii - carcinoma in situ of cervix |
| B58y211 | Secondary cancer of the cervix |
| B41..11 | Cervical carcinoma (uterus) |
| B41yz00 | Malignant neoplasm of other site of cervix nos |
| B410.00 | Malignant neoplasm of endocervix |
| B41..00 | Malignant neoplasm of cervix uteri |
| B831000 | Carcinoma in situ of endocervix |
| B41y000 | Malignant neoplasm of cervical stump |
| B831.00 | Carcinoma in situ of cervix uteri |
| B41y100 | Malignant neoplasm of squamocolumnar junction of cervix |
| Byufa00 | [x]carcinoma in situ of other parts of cervix |
| B410z00 | Malignant neoplasm of endocervix nos |
| B41y.00 | Malignant neoplasm of other site of cervix |
| B58y200 | Secondary malignant neoplasm of cervix uteri |
| B412.00 | Malignant neoplasm, overlapping lesion of cervix uteri |
| B410000 | Malignant neoplasm of endocervical canal |
| B411.00 | Malignant neoplasm of exocervix |
|  |  |
| **Kidney** |  |
| B493.00 | Malignant neoplasm of anterior wall of urinary bladder |
| Bbsz.00 | [m]mesonephroma nos |
| Bbs2.00 | [m]mesonephroma, malignant |
| B180100 | Malignant neoplasm of perinephric tissue |
| Bb5y.00 | [m]hypernephroid tumour |
| B4a1100 | Malignant neoplasm of ureteropelvic junction |
| Bbs1.00 | [m]mesonephric tumour |
| B4a1000 | Malignant neoplasm of renal calyces |
| B702300 | Warthin's tumour |
| Bbl7300 | [m]mesenchymal nephroblastoma |
| B4a..11 | Renal malignant neoplasm |
| Bb5a011 | [m]grawitz tumour |
| Bblj.00 | [m]clear cell sarcoma of kidney |
| Bbs..00 | [m]mesonephromas |
| Bbl7.00 | [m]mixed and stromal renal neoplasms |
| B4az.00 | Malignant neoplasm of kidney or urinary organs nos |
| Bbl7200 | [m]epithelial nephroblastoma |
| Bb5a000 | [m]renal cell carcinoma |
| B4a0000 | Hypernephroma |
| B580.00 | Secondary malignant neoplasm of kidney |
| B4a0.00 | Malignant neoplasm of kidney parenchyma |
| Bbl7000 | [m]mesoblastic nephroma |
| Bbl7.11 | [m]nephromas and nephroblastomas |
| B4a1.00 | Malignant neoplasm of renal pelvis |
| B4a..00 | Malig neop of kidney and other unspecified urinary organs |
| Bb5a012 | [m]hypernephroma |
| Bbl7z00 | [m]mixed or stromal renal neoplasm nos |
| B4a1z00 | Malignant neoplasm of renal pelvis nos |
| Bbl7112 | [m]wilms' tumour |
| Bbl7100 | [m]nephroblastoma nos |
|  |  |
| **Bladder** |  |
| Bb42.00 | [m]transitional cell carcinoma in situ |
| B581100 | Secondary malignant neoplasm of bladder |
| Bb47.00 | [m]transitional cell carcinoma, spindle cell type |
| B49..00 | Malignant neoplasm of urinary bladder |
| B491.00 | Malignant neoplasm of dome of urinary bladder |
| Bb4a.00 | [m]papillary transitional cell carcinoma |
| B49y000 | Malignant neoplasm, overlapping lesion of bladder |
| Byuc500 | [x]2ndry malignant neoplasm/bladder+oth+unsp urinary organs |
| Ba04.00 | Neoplasm of unspecified nature of bladder |
| B492.00 | Malignant neoplasm of lateral wall of urinary bladder |
| B496.00 | Malignant neoplasm of ureteric orifice |
| B490.00 | Malignant neoplasm of trigone of urinary bladder |
| B495.00 | Malignant neoplasm of bladder neck |
| Bb43.00 | [m]transitional cell carcinoma nos |
| B837.00 | Carcinoma in situ of bladder |
| B49y.00 | Malignant neoplasm of other site of urinary bladder |
| B497.00 | Malignant neoplasm of urachus |
| B49z.00 | Malignant neoplasm of urinary bladder nos |
| B494.00 | Malignant neoplasm of posterior wall of urinary bladder |
| B493.00 | Malignant neoplasm of anterior wall of urinary bladder |
|  |  |
| **Testicular** |  |
| B471.00 | Malignant neoplasm of descended testis |
| B47z.12 | Teratoma of testis |
| B48y100 | Malignant neoplasm of tunica vaginalis |
| B836000 | Carcinoma in situ of testis |
| B470z00 | Malignant neoplasm of undescended testis nos |
| B470100 | Malignant neoplasm of retained testis |
| B470.00 | Malignant neoplasm of undescended testis |
| B471000 | Seminoma of descended testis |
| B47z.00 | Malignant neoplasm of testis nos |
| B471100 | Teratoma of descended testis |
| B47..00 | Malignant neoplasm of testis |
| Bbc0.13 | [m]testicular stromal tumour |
| B470300 | Teratoma of undescended testis |
| Bbq1z00 | [m]seminoma nos |
| B47z.11 | Seminoma of testis |
| B58y600 | Secondary malignant neoplasm of testis |
| B470200 | Seminoma of undescended testis |
| Bbq1100 | [m]spermatocytic seminoma |
| Bbq1000 | [m]seminoma, anaplastic type |
| B470000 | Malignant neoplasm of ectopic testis |
| B471z00 | Malignant neoplasm of descended testis nos |
| Bbq1.00 | [m]seminomas |
|  |  |
| **Head and neck** | |
| B200z00 | Malignant neoplasm of nasal cavities nos |
| B202.00 | Malignant neoplasm of maxillary sinus |
| B81y900 | Carcinoma in situ of sphenoidal sinus |
| B502.00 | Malignant neoplasm of lacrimal gland |
| B507100 | Malignant neoplasm of nasolacrimal duct |
| B810.00 | Carcinoma in situ of larynx |
| B07..00 | Malignant neoplasm of nasopharynx |
| B200.00 | Malignant neoplasm of nasal cavities |
| B300.00 | Malignant neoplasm of bones of skull and face |
| B310100 | Malignant neoplasm of soft tissue of face |
| B021.00 | Malignant neoplasm of submandibular gland |
| Bbb1.11 | [m]warthin's tumour |
| B81y700 | Carcinoma in situ of ethmoidal sinus |
| B81y.11 | Carcinoma in situ of nasal sinuses |
| B201100 | Malignant neoplasm of tympanic cavity |
| B02y.00 | Malignant neoplasm of other major salivary glands |
| B203.00 | Malignant neoplasm of ethmoid sinus |
| B022.00 | Malignant neoplasm of sublingual gland |
| B201300 | Malignant neoplasm of mastoid air cells |
| B334z00 | Malignant neoplasm of scalp or skin of neck nos |
| B810z00 | Carcinoma in situ of larynx nos |
| B213z00 | Malignant neoplasm of laryngeal cartilage nos |
| B81y600 | Carcinoma in situ of maxillary sinus |
| B507z00 | Malignant neoplasm of lacrimal duct nos |
| B310400 | Malignant neoplasm of tarsus of eyelid |
| B310000 | Malignant neoplasm of soft tissue of head |
| B200100 | Malignant neoplasm of nasal conchae |
| B507.00 | Malignant neoplasm of lacrimal duct |
| B62z100 | Unspec malig neop lymphoid/histiocytic lymph node head/neck |
| B300100 | Malignant neoplasm of frontal bone |
| B334100 | Malignant neoplasm of skin of neck |
| B550z00 | Malignant neoplasm of head, neck and face nos |
| B200000 | Malignant neoplasm of cartilage of nose |
| B601100 | Lymphosarcoma of lymph nodes of head, face and neck |
| B300900 | Malignant neoplasm of zygomatic bone |
| B550200 | Malignant neoplasm of nose nos |
| B507000 | Malignant neoplasm of lacrimal sac |
| B020.00 | Malignant neoplasm of parotid gland |
| B62y100 | Malignant lymphoma nos of lymph nodes of head, face and neck |
| B626100 | Mast cell malignancy of lymph nodes of head, face and neck |
| B300000 | Malignant neoplasm of ethmoid bone |
| B02z.00 | Malignant neoplasm of major salivary gland nos |
| B622100 | Sezary's disease of lymph nodes of head, face and neck |
| B550400 | Malignant neoplasm of neck nos |
| B201z00 | Malig neop auditory tube, middle ear, mastoid air cells nos |
| B824z00 | Carcinoma in situ of scalp or skin of neck nos |
| B550000 | Malignant neoplasm of head nos |
| B301.00 | Malignant neoplasm of mandible |
| B600100 | Reticulosarcoma of lymph nodes of head, face and neck |
| B560z00 | Secondary unspec malig neop lymph nodes head/face/neck nos |
| B324.00 | Malignant melanoma of scalp and neck |
| B560000 | Secondary and unspec malig neop of superficial parotid ln |
| B560.00 | Secondary and unspec malig neop lymph nodes head/face/neck |
| B823600 | Carcinoma in situ of skin of jaw |
| B21z.00 | Malignant neoplasm of larynx nos |
| B073200 | Malignant neoplasm posterior margin nasal septum and choanae |
| B204.00 | Malignant neoplasm of frontal sinus |
| B310200 | Malignant neoplasm of soft tissue of neck |
| B20..00 | Malig neop nasal cavities, middle ear and accessory sinuses |
| B81y100 | Carcinoma in situ of nasal cavity |
| Bba4.13 | [m]retinal angle tumour |
| B214.00 | Malignant neoplasm, overlapping lesion of larynx |
| B824100 | Carcinoma in situ of skin of neck |
| B824.00 | Carcinoma in situ of scalp and skin of neck |
| B200300 | Malignant neoplasm of vestibule of nose |
| B331100 | Malignant neoplasm of upper eyelid |
| B550.00 | Malignant neoplasm of head, neck and face |
| B310z00 | Malig neop connective and soft tissue head, face, neck nos |
| B300300 | Malignant neoplasm of nasal bone |
| B81y500 | Carcinoma in situ of mastoid air cells |
| B81y800 | Carcinoma in situ of frontal sinus |
| B205.00 | Malignant neoplasm of sphenoidal sinus |
| B20y.00 | Malig neop other site nasal cavity, middle ear and sinuses |
| B334.00 | Malignant neoplasm of scalp and skin of neck |
| B21..00 | Malignant neoplasm of larynx |
| B206.00 | Malignant neoplasm, overlapping lesion of accessory sinuses |
| B582200 | Secondary malignant neoplasm of skin of neck |
| B213.00 | Malignant neoplasm of laryngeal cartilage |
| B0z2.00 | Malignant neoplasm of laryngopharynx |
| B200200 | Malignant neoplasm of septum of nose |
| B310.00 | Malig neop of connective and soft tissue head, face and neck |
| B21y.00 | Malignant neoplasm of larynx, other specified site |
| B300a00 | Malignant neoplasm of maxilla |
|  |  |
| **Lymphoma** |  |
| Bbj5.00 | [m]hodgkin's disease, lymphocytic depletion, reticular type |
| B616500 | Hodgkin's lymphocytic depletion lymph nodes inguinal and leg |
| B612700 | Hodgkin's sarcoma of spleen |
| B611000 | Hodgkin's granuloma of unspecified site |
| Byud200 | [x]other types of diffuse non-hodgkin's lymphoma |
| Bbv2.00 | [m]angiocentrict-cell lymphoma |
| B621700 | Mycosis fungoides of spleen |
| Bbgf.00 | [m]malignant lymphoma, follicular centre cell, cleaved nos |
| B61z100 | Hodgkin's disease nos of lymph nodes of head, face and neck |
| B620.00 | Nodular lymphoma (brill - symmers disease) |
| B613400 | Hodgkin's, lymphocytic-histiocytic pred axilla and arm |
| Bbgv.00 | [m]malignant lymphoma, small cell, noncleaved, diffuse |
| B602100 | Burkitt's lymphoma of lymph nodes of head, face and neck |
| B602400 | Burkitt's lymphoma of lymph nodes of axilla and upper limb |
| B627400 | Diffuse non-hodgkin's small cleaved cell (diffuse) lymphoma |
| B614400 | Hodgkin's nodular sclerosis of lymph nodes of axilla and arm |
| Bbgb.00 | [m]malignant lymphoma, follicular centre cell nos |
| B624100 | Leukaemic reticuloend of lymph nodes of head, face and neck |
| B62yz00 | Malignant lymphoma nos |
| B614.00 | Hodgkin's disease, nodular sclerosis |
| B627w00 | Unspecified b-cell non-hodgkin's lymphoma |
| B627500 | Diffuse non-hodgkin mixed sml & lge cell (diffuse) lymphoma |
| B62x200 | Peripheral t-cell lymphoma |
| Ayuc600 | [x]hiv disease resulting in other non-hodgkin's lymphoma |
| B612100 | Hodgkin's sarcoma of lymph nodes of head, face and neck |
| B62x.00 | Malignant lymphoma otherwise specified |
| Bbq..00 | [m]burkitt's tumours |
| B620z00 | Nodular lymphoma nos |
| Bbga.00 | [m]malignant lymphoma, centroblastic-centrocytic, diffuse |
| B620100 | Nodular lymphoma of lymph nodes of head, face and neck |
| B627c00 | Follicular non-hodgkin's lymphoma |
| B610200 | Hodgkin's paragranuloma of intrathoracic lymph nodes |
| Byud300 | [x]other specified types of non-hodgkin's lymphoma |
| B621300 | Mycosis fungoides of intra-abdominal lymph nodes |
| Bbqz.00 | [m]burkitt's tumour nos |
| B614100 | Hodgkin's nodular sclerosis of head, face and neck |
| B612600 | Hodgkin's sarcoma of intrapelvic lymph nodes |
| Bbk..00 | [m]lymphomas, nodular or follicular |
| Bbj0.00 | [m]hodgkin's disease nos |
| Bbgc.00 | [m]malignant lymphoma, lymphocytic, well differentiated nos |
| Bbgn.00 | [m]malign lymphoma,lymphocytic,intermediate differn, diffuse |
| B602700 | Burkitt's lymphoma of spleen |
| B622300 | Sezary's disease of intra-abdominal lymph nodes |
| B611800 | Hodgkin's granuloma of lymph nodes of multiple sites |
| B602z00 | Burkitt's lymphoma nos |
| Byude00 | [x]unspecified b-cell non-hodgkin's lymphoma |
| B627300 | Diffuse non-hodgkin's small cell (diffuse) lymphoma |
| Bbq0.00 | [m]burkitt's tumour |
| B62y200 | Malignant lymphoma nos of intrathoracic lymph nodes |
| B620300 | Nodular lymphoma of intra-abdominal lymph nodes |
| B611400 | Hodgkin's granuloma of lymph nodes of axilla and upper limb |
| B61..00 | Hodgkin's disease |
| Bbkz.00 | [m]lymphoma, nodular or follicular nos |
| Bbj4.00 | [m]hodgkin's disease,lymphocytic depletion,diffuse fibrosis |
| B616100 | Hodgkin's lymphocytic depletion of head, face and neck |
| B614700 | Hodgkin's disease, nodular sclerosis of spleen |
| B610400 | Hodgkin's paragranuloma of lymph nodes of axilla and arm |
| Bbgc.11 | [m]lymphocytic lymphoma nos |
| B622200 | Sezary's disease of intrathoracic lymph nodes |
| B627000 | Follicular non-hodgkin's small cleaved cell lymphoma |
| B612300 | Hodgkin's sarcoma of intra-abdominal lymph nodes |
| Bbj6.00 | [m]hodgkin's disease, nodular sclerosis nos |
| B620000 | Nodular lymphoma of unspecified site |
| B611100 | Hodgkin's granuloma of lymph nodes of head, face and neck |
| B611700 | Hodgkin's granuloma of spleen |
| B613000 | Hodgkin's, lymphocytic-histiocytic predominance unspec site |
| B627.00 | Non - hodgkin's lymphoma |
| B613500 | Hodgkin's, lymphocytic-histiocytic pred inguinal and leg |
| B602.00 | Burkitt's lymphoma |
| 4m2..00 | Lymphoma staging system |
| B620500 | Nodular lymphoma of lymph nodes of inguinal region and leg |
| Bbg1000 | [m]malignant lymphoma, diffuse nos |
| B620400 | Nodular lymphoma of lymph nodes of axilla and upper limb |
| Bbg1.00 | [m]malignant lymphoma nos |
| B611200 | Hodgkin's granuloma of intrathoracic lymph nodes |
| Bbk2.00 | [m]malignant lymphoma, centroblastic-centrocytic, follicular |
| Bbk7.00 | [m]malignant lymphoma, centroblastic type, follicular |
| B615100 | Hodgkin's mixed cellularity of lymph nodes head, face, neck |
| B615000 | Hodgkin's disease, mixed cellularity of unspecified site |
| Bbj6100 | [m]hodgkin,s disease, nodular sclerosis, mixed cellularity |
| B61z000 | Hodgkin's disease nos, unspecified site |
| Bbgz.00 | [m]lymphoma, diffuse or nos |
| Bbgs.00 | [m]malignant lymphoma, large cell, cleaved, diffuse |
| B62xx00 | Oth and unspecif peripheral & cutaneous t-cell lymphomas |
| B627100 | Follicular non-hodg mixed sml cleavd & lge cell lymphoma |
| B610300 | Hodgkin's paragranuloma of intra-abdominal lymph nodes |
| B616z00 | Hodgkin's disease, lymphocytic depletion nos |
| Bbj1100 | [m]hodgkin,s disease, lymphocytic predominance, nodular |
| Bbk4.00 | [m]malig lymp, lymphocytic, intermediate different, nodular |
| B627x00 | Diffuse non-hodgkin's lymphoma, unspecified |
| Bbgp.00 | [m]malignant lymphoma, mixed small and large cell, diffuse |
| Bbk0.13 | [m]giant follicular lymphoma |
| B621600 | Mycosis fungoides of intrapelvic lymph nodes |
| B620600 | Nodular lymphoma of intrapelvic lymph nodes |
| B61z700 | Hodgkin's disease nos of spleen |
| B615300 | Hodgkin's mixed cellularity of intra-abdominal lymph nodes |
| B621200 | Mycosis fungoides of intrathoracic lymph nodes |
| B612500 | Hodgkin's sarcoma of lymph nodes of inguinal region and leg |
| Bbja.00 | [m]hodgkin's sarcoma |
| Bbgt.00 | [m]malignant lymphoma, large cell, noncleaved, diffuse |
| Bbk3.00 | [m]malig lymphoma, lymphocytic, well differentiated,nodular |
| B613.00 | Hodgkin's disease, lymphocytic-histiocytic predominance |
| B612800 | Hodgkin's sarcoma of lymph nodes of multiple sites |
| B616200 | Hodgkin's lymphocytic depletion of intrathoracic lymph nodes |
| Bbr2700 | [m]adult t-cell leukaemia/lymphoma |
| Bbge.00 | [m]malignant lymphoma, centrocytic |
| B616600 | Hodgkin's lymphocytic depletion of intrapelvic lymph nodes |
| B613z00 | Hodgkin's, lymphocytic-histiocytic predominance nos |
| Bbg2.11 | [m]non hodgkins lymphoma |
| B622600 | Sezary's disease of intrapelvic lymph nodes |
| Bbv0.00 | [m]monocytoid b-cell lymphoma |
| B6...00 | Malignant neoplasm of lymphatic and haemopoietic tissue |
| B614200 | Hodgkin's nodular sclerosis of intrathoracic lymph nodes |
| B612400 | Hodgkin's sarcoma of lymph nodes of axilla and upper limb |
| Bbgg.12 | [m]lymphoblastic lymphoma nos |
| B614600 | Hodgkin's nodular sclerosis of intrapelvic lymph nodes |
| Bbj8.00 | [m]hodgkin's paragranuloma |
| B602800 | Burkitt's lymphoma of lymph nodes of multiple sites |
| B614500 | Hodgkin's nodular sclerosis of inguinal region and leg |
| Byud000 | [x]other hodgkin's disease |
| B622800 | Sezary's disease of lymph nodes of multiple sites |
| B62y.00 | Malignant lymphoma nos |
| B611600 | Hodgkin's granuloma of intrapelvic lymph nodes |
| B610800 | Hodgkin's paragranuloma of lymph nodes of multiple sites |
| B627200 | Follicular non-hodgkin's large cell lymphoma |
| Bbgd.00 | [m]malig lymphoma, lymphocytic, intermediate different nos |
| Bbk6.00 | [m]malig lymp, lymphocytic, poorly differentiated, nodular |
| B627700 | Diffuse non-hodgkin's lymphoblastic (diffuse) lymphoma |
| Bbj2.00 | [m]hodgkin's disease, mixed cellularity |
| B602200 | Burkitt's lymphoma of intrathoracic lymph nodes |
| Byudc00 | [x]diffuse non-hodgkin's lymphoma, unspecified |
| B62x000 | T-zone lymphoma |
| B611500 | Hodgkin's granuloma lymph nodes of inguinal region and leg |
| Byud100 | [x]other types of follicular non-hodgkin's lymphoma |
| B621400 | Mycosis fungoides of lymph nodes of axilla and upper limb |
| B62y300 | Malignant lymphoma nos of intra-abdominal lymph nodes |
| B612000 | Hodgkin's sarcoma of unspecified site |
| Bbjz.00 | [m]hodgkin's disease nos |
| Bblz.00 | [m]mycosis fungoides nos |
| Bbg3.00 | [m]malignant lymphoma, undifferentiated cell type nos |
| B62y800 | Malignant lymphoma nos of lymph nodes of multiple sites |
| Bbj3.00 | [m]hodgkin's disease, lymphocytic depletion nos |
| Bbgg.00 | [m]malignant lymphoma, lymphocytic, poorly different nos |
| Bbgl.00 | [m]malignant lymphoma, small lymphocytic nos |
| B610600 | Hodgkin's paragranuloma of intrapelvic lymph nodes |
| B621800 | Mycosis fungoides of lymph nodes of multiple sites |
| B616700 | Hodgkin's disease, lymphocytic depletion of spleen |
| Bbj7.00 | [m]hodgkin's disease, nodular sclerosis, cellular phase |
| B614300 | Hodgkin's nodular sclerosis of intra-abdominal lymph nodes |
| B627d00 | Diffuse non-hodgkin's centroblastic lymphoma |
| B602600 | Burkitt's lymphoma of intrapelvic lymph nodes |
| B621100 | Mycosis fungoides of the lymph nodes of head, face and neck |
| Bbmd.00 | [m] cutaneous lymphoma |
| B610100 | Hodgkin's paragranuloma of lymph nodes of head, face, neck |
| B622000 | Sezary's disease of unspecified site |
| B602500 | Burkitt's lymphoma of lymph nodes of inguinal region and leg |
| Bbk0.11 | [m]brill - symmers' disease |
| Byudf00 | [x]non-hodgkin's lymphoma, unspecified type |
| B62x100 | Lymphoepithelioid lymphoma |
| B616400 | Hodgkin's lymphocytic depletion lymph nodes axilla and arm |
| Bbj1000 | [m]hodgkin,s disease, lymphocytic predominance, diffuse |
| B622700 | Sezary's disease of spleen |
| Bbgk.00 | [m]malig lymphoma, follicular centre cell, non-cleaved nos |
| B61z200 | Hodgkin's disease nos of intrathoracic lymph nodes |
| Bbgj.00 | [m]malignant lymphoma, centroblastic type nos |
| Bbg2.00 | [m]malignant lymphoma, non hodgkin's type |
| B61z300 | Hodgkin's disease nos of intra-abdominal lymph nodes |
| Bbb1.00 | [m]adenolymphoma |
| Bbgm.00 | [m]malignant lymphoma, small cleaved cell, diffuse |
| B62y000 | Malignant lymphoma nos of unspecified site |
| B6z..00 | Malignant neoplasm lymphatic or haematopoietic tissue nos |
| Bbm5.00 | [m] peripheral t-cell lymphoma nos |
| Bbg8.00 | [m]malignant lymphoma, immunoblastic type |
| B621000 | Mycosis fungoides of unspecified site |
| B615.00 | Hodgkin's disease, mixed cellularity |
| Bbj..00 | [m]hodgkin's disease |
| B612z00 | Hodgkin's sarcoma nos |
| B616.00 | Hodgkin's disease, lymphocytic depletion |
| Bbl1.00 | [m]sezary's disease |
| B61z500 | Hodgkin's disease nos of lymph nodes inguinal region and leg |
| Byudd00 | [x]oth and unspecif peripheral & cutaneous t-cell lymphomas |
| B621.00 | Mycosis fungoides |
| B610500 | Hodgkin's paragranuloma lymph nodes inguinal region and leg |
| Bbg7.00 | [m]malignant lymphoma, lymphoplasmacytoid type |
| B615600 | Hodgkin's mixed cellularity of intrapelvic lymph nodes |
| B61z800 | Hodgkin's disease nos of lymph nodes of multiple sites |
| B616000 | Hodgkin's lymphocytic depletion of unspecified site |
| B622z00 | Sezary's disease nos |
| Bbj1.00 | [m]hodgkin's disease, lymphocytic predominance |
| B62y400 | Malignant lymphoma nos of lymph nodes of axilla and arm |
| Bbj6200 | [m]hodgkin,s disease, nodular sclerosis, lymphocytic deplet |
| B611.00 | Hodgkin's granuloma |
| B612.00 | Hodgkin's sarcoma |
| Bbl..00 | [m]mycosis fungoides |
| B611z00 | Hodgkin's granuloma nos |
| Byudf11 | [x]non-hodgkin's lymphoma nos |
| B61z400 | Hodgkin's disease nos of lymph nodes of axilla and arm |
| 4m20.00 | Lymphoma stage i |
| B613800 | Hodgkin's, lymphocytic-histiocytic pred of multiple sites |
| B620200 | Nodular lymphoma of intrathoracic lymph nodes |
| A789700 | Hiv dis resulting oth types of non-hodgkin's lymphoma |
| B627800 | Diffuse non-hodgkin's lymphoma undifferentiated (diffuse) |
| B602300 | Burkitt's lymphoma of intra-abdominal lymph nodes |
| B615400 | Hodgkin's mixed cellularity of lymph nodes of axilla and arm |
| B627c11 | Follicular lymphoma nos |
| B616800 | Hodgkin's lymphocytic depletion lymph nodes multiple sites |
| B614800 | Hodgkin's nodular sclerosis of lymph nodes of multiple sites |
| B627600 | Diffuse non-hodgkin's immunoblastic (diffuse) lymphoma |
| Bbgq.00 | [m]malignant lymphomatous polyposis |
| B61z.00 | Hodgkin's disease nos |
| Bbm9.00 | [m] monocytoid b-cell lymphoma |
| B615800 | Hodgkin's mixed cellularity of lymph nodes of multiple sites |
| B613700 | Hodgkin's, lymphocytic-histiocytic predominance of spleen |
| B613200 | Hodgkin's, lymphocytic-histiocytic pred intrathoracic nodes |
| B62x600 | True histiocytic lymphoma |
| B610.00 | Hodgkin's paragranuloma |
| B620700 | Nodular lymphoma of spleen |
| B62y500 | Malignant lymphoma nos of lymph node inguinal region and leg |
| B610700 | Hodgkin's paragranuloma of spleen |
| B61z600 | Hodgkin's disease nos of intrapelvic lymph nodes |
| B610z00 | Hodgkin's paragranuloma nos |
| Bbgr.00 | [m]malignant lymphoma, large cell, diffuse nos |
| B622.00 | Sezary's disease |
| Bbg5.00 | [m]malignant lymphoma, convoluted cell type nos |
| B615700 | Hodgkin's disease, mixed cellularity of spleen |
| B613300 | Hodgkin's, lymphocytic-histiocytic pred intra-abdominal node |
| B613600 | Hodgkin's, lymphocytic-histiocytic pred intrapelvic nodes |
| B614000 | Hodgkin's disease, nodular sclerosis of unspecified site |
| B611300 | Hodgkin's granuloma of intra-abdominal lymph nodes |
| Bbg1.11 | [m]lymphoma nos |
| Bbg4.00 | [m]malignant lymphoma, stem cell type |
| Bbm4.00 | [m]true histiocytic lymphoma |
| Bbj9.00 | [m]hodgkin's granuloma |
| 4m23.00 | Lymphoma stage iv |
| B621z00 | Mycosis fungoides nos |
| B61zz00 | Hodgkin's disease nos |
| B612200 | Hodgkin's sarcoma of intrathoracic lymph nodes |
| A789600 | Hiv disease resulting in burkitt's lymphoma |
| Bbl0.00 | [m]mycosis fungoides |
| B614z00 | Hodgkin's disease, nodular sclerosis nos |
| B610000 | Hodgkin's paragranuloma of unspecified site |
| B62y700 | Malignant lymphoma nos of spleen |
| Bbg..00 | [m]lymphomas, nos or diffuse |
| B621500 | Mycosis fungoides of lymph nodes of inguinal region and leg |
| B613100 | Hodgkin's, lymphocytic-histiocytic pred of head, face, neck |
| Bbk1.00 | [m]malig lymphoma, mixed lymphocytic-histiocytic, nodular |
| Bbj6000 | [m]hodgkin,s disease, nodular sclerosis, lymphocytic predom |
| B627b00 | Other types of follicular non-hodgkin's lymphoma |
| Bbg9.00 | [m]malignant lymphoma, mixed lymphocytic-histiocytic nos |
| Bbk0.00 | [m]malignant lymphoma, nodular nos |
| B615500 | Hodgkin's mixed cellularity of lymph nodes inguinal and leg |
| B62y600 | Malignant lymphoma nos of intrapelvic lymph nodes |
| B622500 | Sezary's disease of lymph nodes of inguinal region and leg |
| B616300 | Hodgkin's lymphocytic depletion intra-abdominal lymph nodes |
| B615200 | Hodgkin's mixed cellularity of intrathoracic lymph nodes |
| B615z00 | Hodgkin's disease, mixed cellularity nos |
| Bbmh.00 | [m] large cell lymphoma |
| B620800 | Nodular lymphoma of lymph nodes of multiple sites |
| B602000 | Burkitt's lymphoma of unspecified site |
| B62y100 | Malignant lymphoma nos of lymph nodes of head, face and neck |
| B622100 | Sezary's disease of lymph nodes of head, face and neck |
|  |  |
| **Leukaemia** |  |
| Bbr8.00 | [m]eosinophilic leukaemias |
| Bbr3z00 | [m]plasma cell leukaemia nos |
| B64..00 | Lymphoid leukaemia |
| Bbr9012 | [m]schilling-type monocytic leukaemia |
| B66yz00 | Other monocytic leukaemia nos |
| B64y000 | Aleukaemic lymphoid leukaemia |
| Bbr3000 | [m]plasma cell leukaemia |
| Bbr4111 | [m]di guglielmo's disease |
| Bbra800 | [m]leukaemic reticuloendotheliosis |
| Bbr6011 | [m]granulocytic leukaemia nos |
| B651.00 | Chronic myeloid leukaemia |
| B660.00 | Acute monocytic leukaemia |
| B642.00 | Subacute lymphoid leukaemia |
| Bbra.00 | [m]miscellaneous leukaemias |
| Bbr6.00 | [m]myeloid leukaemias |
| Bbr6700 | [m]acute myelomonocytic leukaemia |
| Bbr8000 | [m]eosinophilic leukaemia |
| B65y.00 | Other myeloid leukaemia |
| Bbr9400 | [m]aleukaemic monocytic leukaemia |
| B66..11 | Histiocytic leukaemia |
| Bbr2400 | [m]aleukaemic lymphoid leukaemia |
| Bbra100 | [m]megakaryocytic leukaemia |
| B66y.00 | Other monocytic leukaemia |
| B67y.00 | Other and unspecified leukaemia |
| B64..11 | Lymphatic leukaemia |
| Byud600 | [x]other myeloid leukaemia |
| B68..00 | Leukaemia of unspecified cell type |
| Bbr4z00 | [m]erythroleukaemia nos |
| Bbr6500 | [m]neutrophilic leukaemia |
| B624800 | Leukaemic reticuloend of lymph nodes of multiple sites |
| Bbra500 | [m]acute megakaryoblastic leukaemia |
| Bbr0111 | [m]blast cell leukaemia |
| B64yz00 | Other lymphoid leukaemia nos |
| Bbr9200 | [m]subacute monocytic leukaemia |
| Byud700 | [x]other monocytic leukaemia |
| Bbr5.00 | [m]lymphosarcoma cell leukaemias |
| Bbr0300 | [m]chronic leukaemia nos |
| B662.00 | Subacute monocytic leukaemia |
| B66z.00 | Monocytic leukaemia nos |
| Bbr6311 | [m]naegeli-type monocytic leukaemia |
| B624700 | Leukaemic reticuloendotheliosis of spleen |
| Bbr1000 | [m]compound leukaemia |
| Bbr2500 | [m]prolymphocytic leukaemia |
| Bbr2011 | [m]lymphatic leukaemia |
| B65y000 | Aleukaemic myeloid leukaemia |
| Bbrz.00 | [m]leukaemia nos |
| B64y.00 | Other lymphoid leukaemia |
| B624200 | Leukaemic reticuloendotheliosis of intrathoracic lymph nodes |
| Byud500 | [x]other lymphoid leukaemia |
| Bbr1.00 | [m]compound leukaemias |
| B651200 | Chronic neutrophilic leukaemia |
| Bbr1011 | [m]mixed leukaemia |
| B661.00 | Chronic monocytic leukaemia |
| B64y200 | Adult t-cell leukaemia |
| B66..12 | Monoblastic leukaemia |
| Bbr0200 | [m]subacute leukaemia nos |
| Bbr1z00 | [m]compound leukaemia nos |
| Bbr3.00 | [m]plasma cell leukaemias |
| Bbr2.00 | [m]lymphoid leukaemias |
| Bbr7.00 | [m]basophilic leukaemias |
| B624400 | Leukaemic reticuloend of lymph nodes of axilla and arm |
| B64z.00 | Lymphoid leukaemia nos |
| B624z00 | Leukaemic reticuloendotheliosis nos |
| B690.00 | Acute myelomonocytic leukaemia |
| Bbr9z00 | [m]other monocytic leukaemia nos |
| B68y.00 | Other leukaemia of unspecified cell type |
| B651000 | Chronic eosinophilic leukaemia |
| Bbr8z00 | [m]eosinophilic leukaemia nos |
| B650.00 | Acute myeloid leukaemia |
| Bbr0000 | [m]leukaemia nos |
| B624.12 | Hairy cell leukaemia |
| B67z.00 | Other specified leukaemia nos |
| Bbr4000 | [m]erythroleukaemia |
| B651z00 | Chronic myeloid leukaemia nos |
| Bbr9100 | [m]acute monocytic leukaemia |
| B66..00 | Monocytic leukaemia |
| Bbr0113 | [m]stem cell leukaemia |
| Bbr4.00 | [m]erythroleukaemias |
| Bbr2300 | [m]chronic lymphoid leukaemia |
| Bbr..00 | [m]leukaemias |
| B65y100 | Acute promyelocytic leukaemia |
| Bbr9.00 | [m]monocytic leukaemias |
| Bbr9000 | [m]monocytic leukaemia nos |
| Bbraz00 | [m]miscellaneous leukaemia nos |
| Bbr0400 | [m]aleukaemic leukaemia nos |
| Bbr7z00 | [m]basophilic leukaemia nos |
| Bbr9011 | [m]histiocytic leukaemia |
| Bbr2200 | [m]subacute lymphoid leukaemia |
| Bbr6800 | [m]chronic myelomonocytic leukaemia |
| B624600 | Leukaemic reticuloendotheliosis of intrapelvic lymph nodes |
| Bbr6200 | [m]subacute myeloid leukaemia |
| B681.00 | Chronic leukaemia nos |
| Byud800 | [x]other specified leukaemias |
| Bbra000 | [m]mast cell leukaemia |
| B691.00 | Chronic myelomonocytic leukaemia |
| B682.00 | Subacute leukaemia nos |
| Bbr7000 | [m]basophilic leukaemia |
| Bbr5000 | [m]lymphosarcoma cell leukaemia |
| Bbr2100 | [m]acute lymphoid leukaemia |
| Bbr6600 | [m]acute promyelocytic leukaemia |
| Bbr2600 | [m]burkitt's cell leukaemia |
| Bbr6400 | [m]aleukaemic myeloid leukaemia |
| Bbr6000 | [m]myeloid leukaemia nos |
| B67yz00 | Other and unspecified leukaemia nos |
| B624300 | Leukaemic reticuloend of intra-abdominal lymph nodes |
| Bbr5z00 | [m]lymphosarcoma cell leukaemia nos |
| Bbr0112 | [m]blastic leukaemia |
| B624.11 | Leukaemic reticuloendotheliosis |
| B692.00 | Subacute myelomonocytic leukaemia |
| B67y000 | Lymphosarcoma cell leukaemia |
| Bbr0100 | [m]acute leukaemia nos |
| B624.00 | Leukaemic reticuloendotheliosis |
| B652.00 | Subacute myeloid leukaemia |
| Byud900 | [x]other leukaemia of unspecified cell type |
| B68z.00 | Leukaemia nos |
| B640.00 | Acute lymphoid leukaemia |
| B670.00 | Acute erythraemia and erythroleukaemia |
| B64y100 | Prolymphocytic leukaemia |
| B624500 | Leukaemic reticuloend of lymph nodes inguinal region and leg |
| Bbra111 | [m]thrombocytic leukaemia |
| B67..00 | Other specified leukaemia |
| B65..00 | Myeloid leukaemia |
| Bbr0.00 | [m]leukaemias unspecified |
| B66y000 | Aleukaemic monocytic leukaemia |
| Bbr9300 | [m]chronic monocytic leukaemia |
| B680.00 | Acute leukaemia nos |
| B673.00 | Mast cell leukaemia |
| B651.11 | Chronic granulocytic leukaemia |
| B631.00 | Plasma cell leukaemia |
| Bbr6300 | [m]chronic myeloid leukaemia |
| B641.00 | Chronic lymphoid leukaemia |
| Bbr2000 | [m]lymphoid leukaemia nos |
| Bbr6100 | [m]acute myeloid leukaemia |
| B65yz00 | Other myeloid leukaemia nos |
| Bbr0z00 | [m]leukaemia unspecified, nos |
| B672.11 | Thrombocytic leukaemia |
| B672.00 | Megakaryocytic leukaemia |
| Bbra400 | [m]hairy cell leukaemia |
| Bbr6z00 | [m]other myeloid leukaemia nos |
| B624000 | Leukaemic reticuloendotheliosis of unspecified sites |
| Bbr2z00 | [m]other lymphoid leukaemia nos |
| B69..00 | Myelomonocytic leukaemia |
| B65z.00 | Myeloid leukaemia nos |
| B641.11 | Chronic lymphatic leukaemia |
| Bbra411 | [m]leukaemic reticuloendotheliosis |
| B624100 | Leukaemic reticuloend of lymph nodes of head, face and neck |
| Bbr2700 | [m]adult t-cell leukaemia/lymphoma |
|  |  |
| **Myeloma** |  |
| Bbn3.00 | [m]plasma cell tumour, malignant |
| Bbmk.00 | [m]waldenstrom's macroglobulinaemia |
| Bbn..00 | [m]plasma cell tumours |
| Bbn0.00 | [m]plasma cell myeloma |
| B630000 | Malignant plasma cell neoplasm, extramedullary plasmacytoma |
| B936.11 | Myeloma - solitary |
| B630.00 | Multiple myeloma |
| B63..00 | Multiple myeloma and immunoproliferative neoplasms |
| B630200 | Plasmacytoma nos |
| Bbmc.00 | [m] t-gamma lymphoproliferative disease |
| Bbm6.00 | [m] alpha heavy chain disease |
| Bbn2.00 | [m]plasmacytoma nos |
| B630100 | Solitary myeloma |
| Bbnz.00 | [m]plasma cell tumour nos |
| B936.12 | Plasmacytoma nos |
| Bbn2.11 | [m]monostotic myeloma |
| B63z.00 | Immunoproliferative neoplasm or myeloma nos |
| Bbn2.12 | [m]solitary myeloma |
| Bbme.00 | [m] gamma heavy chain disease |
| B630300 | Lambda light chain myeloma |
| Bbn0.11 | [m]multiple myeloma |
| Bbn0.14 | [m]plasmacytic myeloma |
| Bbn0.13 | [m]myelomatosis |
| Bbn0.12 | [m]myeloma nos |
| B630.12 | Myelomatosis |
