# Supplementary material for: Age and Gender Variations in Cancer Diagnostic Intervals in 15 Cancers: Analysis of Data from the UK Clinical Practice Research Datalink
Source: PLoS One. 2015 May 15;10(5):e0127717. doi: 10.1371/journal.pone.0127717 (PMC4433335; doi:10.1371/journal.pone.0127717)
Supplement: S2 Table — (DOCX) [file pone.0127717.s002.docx]

**S2 Table. Cancer site specific symptom Read Codes**

**Symptoms are listed in an alphabetical order as many symptoms were used in multiple cancers**

| **Read code** | **Read term** | |  |
| --- | --- | --- | --- |
| **Abdominal pain** |  | |  |
| 1969.00 | Abdominal pain | |  |
| 1972.00 | Epigastric pain | |  |
| R090500 | [D]Epigastric pain | |  |
| R090.00 | [D]Abdominal pain | |  |
| 196..11 | Abdominal pain type | |  |
| R090E00 | [D]Recurrent acute abdominal pain | |  |
| R090z00 | [D]Abdominal pain NOS | |  |
| 197B.00 | Upper abdominal pain | |  |
| 1971.00 | Central abdominal pain | |  |
| R090600 | [D]Umbilical pain | |  |
| 1963.00 | Non-colicky abdominal pain | |  |
| 197..13 | Site of abdominal pain | |  |
| 196..12 | Type of GIT pain - symptom | |  |
| R090G12 | [D] Perineal pain | |  |
| 1979.00 | Suprapubic pain | |  |
| R090J00 | [D]Right upper quadrant pain | |  |
| 1962.00 | Colicky abdominal pain | |  |
| R090K00 | [D]Left upper quadrant pain | |  |
| R090H00 | [D]Upper abdominal pain | |  |
| R090L00 | [D]Left lower quadrant pain | |  |
| 197D.00 | Right upper quadrant pain | |  |
| R090G00 | [D]Pelvic and perineal pain | |  |
| 197A.11 | General abdominal pain-symptom | |  |
| 25C2.00 | O/E - abd.pain-R.hypochondrium | |  |
| R073.00 | [D]Flatulence, eructation and gas pain | |  |
| 25CZ.00 | O/E -abd.pain on palpation NOS | |  |
| 196..00 | Type of GIT pain | |  |
| 25C..00 | O/E - abdo. pain on palpation | |  |
| 25C3.00 | O/E - abd. pain - epigastrium | |  |
| R090N00 | [D]Nonspecific abdominal pain | |  |
| R090M00 | [D]Right lower quadrant pain | |  |
| R090800 | [D]Suprapubic pain | |  |
| 25C..11 | O/E - epigastric pain on palp. | |  |
| R079.00 | [D] Defaecation painful | |  |
| 197C.00 | Lower abdominal pain | |  |
| 25C6.00 | O/E - abd. pain - umbilical | |  |
| 197A.00 | Generalised abdominal pain | |  |
| 197..00 | Site of GIT pain | |  |
| 25C7.00 | O/E - abd. pain - L.lumbar | |  |
| R073200 | [D]Gas pain (abdominal) | |  |
| 1969000 | Abdominal wall pain | |  |
| 1A5A.00 | C/O perineal pain | |  |
| R090y00 | [D]Other specified abdominal pain | |  |
| 25C5.00 | O/E - abd. pain - R.lumbar | |  |
| 25C4.00 | O/E - abd.pain-L.hypochondrium | |  |
| R073z00 | [D]Flatulence, eructation and gas pain NOS | |  |
| Ryu1100 | [X]Other and unspecified abdominal pain | |  |
|  |  | |  |
| **Bleeding** |  | |  |
| R047.11 | [D]Nosebleed | |  |
| R09z000 | [D]Umbilical bleeding | |  |
| Kyu9D00 | [X]Other specified abnormal uterine and vaginal bleeding | |  |
| K5Ez.00 | Abnormal uterine and vaginal bleeding, unspecified | |  |
| K5E0.00 | Abnormal uterine bleeding unrelated to menstrual cycle | |  |
| K5E1.00 | Abnormal uterine bleeding, unspecified | |  |
| K5E2.00 | Abnormal vaginal bleeding, unspecified | |  |
| L11..12 | Antepartum bleeding | |  |
| K56y111 | Bleeding - vaginal NOS | |  |
| J110111 | Bleeding acute gastric ulcer | |  |
| J121111 | Bleeding chronic duodenal ulcer | |  |
| J111111 | Bleeding chronic gastric ulcer | |  |
| D30..11 | Bleeding disorders | |  |
| J510900 | Bleeding diverticulosis | |  |
| SP03216 | Bleeding due to intrauterine contraceptive device | |  |
| K19y400 | Bleeding from urethra | |  |
| 1928.00 | Bleeding gums | |  |
| G848000 | Bleeding haemorrhoids NOS | |  |
| L10y.11 | Bleeding in early pregnancy | |  |
| L36..11 | Bleeding postpartum | |  |
| J573.11 | Bleeding PR | |  |
| 25T0.00 | Bleeding stoma | |  |
| 42Q..11 | Bleeding tests | |  |
| 4762.11 | Blood in faeces | |  |
| 19E6.00 | Blood in faeces | |  |
| 19E6.11 | Blood in faeces symptom | |  |
| 172..00 | Blood in sputum - haemoptysis | |  |
| 172..11 | Blood in sputum - symptom | |  |
| J681.11 | Blood in stool | |  |
| J681.13 | Blood in stools altered | |  |
| 4B34.11 | Blood in synovial fluid | |  |
| 1A45.00 | Blood in urine - haematuria | |  |
| 1A45.11 | Blood in urine - symptom | |  |
| 1994.11 | Blood in vomit - symptom | |  |
| 4A23.11 | Blood in vomit O/E | |  |
| 19EG.00 | Blood on pants | |  |
| 19ED.00 | Blood on toilet paper | |  |
| K56y112 | BPV - Vaginal bleeding | |  |
| K59z.11 | Break - through bleeding | |  |
| 158..11 | C/O p.v. bleeding | |  |
| D3...00 | Clotting and bleeding disorders | |  |
| D3z..00 | Clotting or bleeding disorder NOS | |  |
| 42QZ.00 | Coag./bleeding test NOS | |  |
| 42Q2.00 | Coag./bleeding tests abnormal | |  |
| K587.00 | Contact bleeding of cervix | |  |
| SP03217 | Contraception IUCD causing bleeding | |  |
| K59yx11 | Dysfunctional uterine bleeding | |  |
| G845000 | External bleeding haemorrhoids | |  |
| J68z.11 | GIB - Gastrointestinal bleeding | |  |
| 158..00 | H/O: abnormal uterine bleeding | |  |
| 1455.11 | H/O: bleeding disorder | |  |
| 14CA.11 | H/O: GI Bleed | |  |
| 1582.00 | H/O: inter-menstrual bleeding | |  |
| 1581.00 | H/O: post-coital bleeding | |  |
| 1583.00 | H/O: post-menopausal bleeding | |  |
| 14CD.11 | H/O: upper GIT bleed | |  |
| 158Z.00 | H/O:abnormal uterine bleed NOS | |  |
| 1C62.00 | Has nose bleeds - epistaxis | |  |
| 1584.00 | Heavy episode of vaginal bleeding | |  |
| 66UI.00 | Hormone replacement therapy bleed pattern - abnormal | |  |
| 66UK.00 | Hormone replacement therapy bleed pattern - no bleeding | |  |
| K59y300 | Intermenstrual bleeding | |  |
| K596.11 | Intermenstrual bleeding - irregular | |  |
| K595.11 | Intermenstrual bleeding - regular | |  |
| G842000 | Internal bleeding haemorrhoids | |  |
| K599.00 | Mid-cycle bleeding | |  |
| 1C6..00 | Nose bleed symptom | |  |
| 1C6Z.00 | Nose bleed symptom NOS | |  |
| 2556.00 | O/E - bleeding gums | |  |
| 2693.00 | O/E-speculum=bleeding thro' os | |  |
| G850.00 | Oesophageal varices with bleeding | |  |
| G852000 | Oesophageal varices with bleeding in diseases EC | |  |
| G851.00 | Oesophageal varices without bleeding | |  |
| K5E..00 | Other abnormal uterine and vaginal bleeding | |  |
| 196B.00 | Painful rectal bleeding | |  |
| 196C.00 | Painless rectal bleeding | |  |
| Q41y111 | Perinatal transient vaginal bleeding | |  |
| K597.00 | Postcoital bleeding | |  |
| K5A1.00 | Postmenopausal bleeding | |  |
| K59B.00 | Postmenopausal postcoital bleeding | |  |
| J573012 | PRB - Rectal bleeding | |  |
| K59A.00 | Premenopausal postcoital bleeding | |  |
| K593.11 | Pubertal bleeding and menorrhagia | |  |
| K593.00 | Puberty bleeding | |  |
| J573011 | Rectal bleeding | |  |
| K19y411 | Urethral bleeding | |  |
| 158..12 | Vaginal bleeding | |  |
|  |  | |  |
| **Bone pain** |  | |  |
| R090G11 | [D] Pelvic pain | |  |
| R065011 | [D] Retrosternal chest pain | |  |
| R01z100 | [D]Growing pains - limbs | |  |
| R090700 | [D]Hypochondrial pain | |  |
| R040z11 | [D]Jaw pain | |  |
| R065A00 | [D]Musculoskeletal chest pain | |  |
| R01z200 | [D]Musculoskeletal pain | |  |
| R090A00 | [D]Pain in left iliac fossa | |  |
| R090900 | [D]Pain in right iliac fossa | |  |
| R065900 | [D]Parasternal chest pain | |  |
| R090G00 | [D]Pelvic and perineal pain | |  |
| 1DC2.00 | Aching pain | |  |
| N12..13 | Acute back pain - disc | |  |
| N142.13 | Acute back pain - lumbar | |  |
| N141.11 | Acute back pain - thoracic | |  |
| N145.11 | Acute back pain - unspecified | |  |
| N094711 | Ankle joint pain | |  |
| N245.11 | Ankle pain | |  |
| 1M13.00 | Ankle pain | |  |
| N094W00 | Anterior knee pain | |  |
| 1M12.00 | Anterior knee pain | |  |
| N245.12 | Arm pain | |  |
| N245500 | Axillary pain | |  |
| N33A.00 | Bone pain | |  |
| N33A000 | Bony pelvic pain | |  |
| 1A53.12 | C/O - lumbar pain | |  |
| 1D13111 | C/O - pain in big toe | |  |
| 1D13100 | C/O - pain in hallux | |  |
| 1D13000 | C/O - pain in toes | |  |
| N245400 | Calf pain | |  |
| 1822.00 | Central chest pain | |  |
| N131.00 | Cervicalgia - pain in neck | |  |
| N33A100 | Clavicle pain | |  |
| 182B000 | Costal margin chest pain | |  |
| N094211 | Elbow joint pain | |  |
| N094D11 | Elbow joint pain | |  |
| 1M00.11 | Elbow pain | |  |
| N245012 | Finger pain | |  |
| N094411 | Hand joint pain | |  |
| N245.14 | Hand pain | |  |
| N245000 | Hand pain | |  |
| N245.15 | Heel pain | |  |
| N094512 | Hip joint pain | |  |
| N094K12 | Hip pain | |  |
| 197..12 | Iliac fossa pain | |  |
| N094611 | Knee joint pain | |  |
| 1M10.00 | Knee pain | |  |
| 1978.00 | Left iliac fossa pain | |  |
| 1973.00 | Left subcostal pain | |  |
| N096.12 | Musculoskeletal pain - joints | |  |
| SP07A00 | Pain due to hip joint prosthesis | |  |
| SP07B00 | Pain due to knee joint prosthesis | |  |
| SP07C00 | Pain due to shoulder joint prosthesis | |  |
| N245300 | Pain in arm | |  |
| N245.19 | Pain in buttock | |  |
| N131.11 | Pain in cervical spine | |  |
| N147211 | Pain in coccyx | |  |
| 1M00.00 | Pain in elbow | |  |
| N094.00 | Pain in joint - arthralgia | |  |
| N245200 | Pain in leg | |  |
| N245.00 | Pain in limb | |  |
| 1M1..00 | Pain in lower limb | |  |
| N142.00 | Pain in lumbar spine | |  |
| 1977.00 | Right iliac fossa pain | |  |
| 1974.00 | Right subcostal pain | |  |
| N094111 | Shoulder joint pain | |  |
| N245.17 | Shoulder pain | |  |
| N245700 | Shoulder pain | |  |
| 197..14 | Subcostal pain | |  |
| J046400 | Temporomandibular joint-pain-dysfunction syndrome | |  |
| N245.18 | Thigh pain | |  |
| N245011 | Thumb pain | |  |
| N245111 | Toe pain | |  |
| N094311 | Wrist joint pain | |  |
| N094F11 | Wrist pain | |  |
|  |  | |  |
| **Breast lump** |  | |  |
| K317100 | Lump in breast | |  |
| 1A8..00 | Breast lump symptom | |  |
| 6862.00 | Breast neoplasm screen | |  |
| R022200 | [D]Lump, localized and superficial | |  |
| 2I11.00 | O/E - a lump | |  |
| 9OHE.00 | Patient breast aware | |  |
| R022.00 | [D]Local superficial swelling, mass or lump | |  |
| 7131211 | Lumpectomy of breast | |  |
| K300.00 | Solitary cyst of breast | |  |
| R022700 | [D]Axillary lump | |  |
| 1596.00 | H/O: breast problem | |  |
| R066100 | [D]Chest lump | |  |
| 1A82.00 | Breast lump present | |  |
| 26B7.12 | Lumpy breasts | |  |
| 26E..11 | O/E - breast lump position | |  |
| 1A8Z.00 | Breast lump symptom NOS | |  |
| R066.00 | [D]Swelling, mass and lump of chest | |  |
| 1A86.00 | Breast lump detected by self-examination | |  |
| 26F..00 | O/E - breast lump size | |  |
| 26E..00 | O/E - breast lump palpated | |  |
| 1A83.00 | Breast lump detected by clinician examination | |  |
| 26G..00 | O/E - breast lump consistency | |  |
| 26E5.00 | O/E-breast lump-upper out-quad | |  |
| 7131300 | Wire guided excision of breast lump under radiolog control | |  |
| R022z00 | [D]Local superficial swelling, mass or lump NOS | |  |
| 1A84.00 | Breast lump detected by mammogram | |  |
| 7131B11 | Lumpectomy NEC | |  |
| 7131600 | Wire guided wide local excision breast lump radiolog control | |  |
| R022600 | [D]Localized swelling, mass and lump, multiple sites | |  |
| 26H..11 | O/E - breast lump - outline | |  |
| 26I..00 | O/E - breast lump tethering | |  |
| 26E2.00 | O/E-breast lump-nipple/central | |  |
| 26F1.00 | O/E - breast lump - pea size | |  |
| 26E3.00 | O/E-breast lump-upper in-quad | |  |
| 26G2.00 | O/E - breast lump cystic | |  |
| 26EZ.00 | O/E - breast lump palpated NOS | |  |
| 26E6.00 | O/E-breast lump-lower out-quad | |  |
| 26E4.00 | O/E -breast lump-lower in-quad | |  |
| 26E7.00 | O/E-breast lump- axillary tail | |  |
| 7N12100 | [SO]Upper outer quadrant of breast | |  |
| 26G3.00 | O/E - breast lump hard | |  |
| 26F2.00 | O/E - breast lump - plum size | |  |
| 26G1.00 | O/E - breast lump soft | |  |
| 1A85.00 | Breast lump detected by partner | |  |
| R066z00 | [D]Swelling, mass or lump of chest NOS | |  |
| 26FZ.00 | O/E - breast lump size NOS | |  |
| 26GZ.00 | O/E - breast lump consist. NOS | |  |
| 26BB.00 | Contour of breast distorted | |  |
| 26H..00 | O/E - breast lump regularity | |  |
| 26H1.00 | O/E - breast lump smooth | |  |
| 26H2.00 | O/E - breast lump irregular | |  |
| 26I1.00 | O/E - breast lump not tethered | |  |
| 26IZ.00 | O/E - breast lump tethered NOS | |  |
| 26I2.00 | O/E -breast lump fixed to skin | |  |
| 26F3.00 | O/E-breast lump-tangerine size | |  |
| 26F4.00 | O/E - breast lump-orange size | |  |
| 26HZ.00 | O/E-breast lump regularity NOS | |  |
|  |  | |  |
| **Breast pain** |  | |  |
| K317000 | Mastodynia - pain in breast | |  |
| 1596.00 | H/O: breast problem | |  |
| 26BH.00 | Breast tenderness | |  |
| K317z00 | Breast signs and symptoms NOS | |  |
| K317011 | Breast soreness | |  |
| 7N12.00 | [SO]Breast | |  |
| 1424.00 | H/O: * breast | |  |
| K317.00 | Breast signs and symptoms | |  |
| 26BD.00 | Intractable breast pain | |  |
| Kyu7100 | [X]Other signs and symptoms in breast | |  |
|  |  | |  |
| **Breast skin changes** |  | |  |
| 26C4.00 | Nipple eczema | |  |
| 7N72000 | [SO]Skin of breast | |  |
| 26B4.00 | O/E - peau d'orange | |  |
| 26C4.00 | Nipple eczema | |  |
|  |  | |  |
| **Bruising** |  | |  |
| SE...00 | Contusion (bruise) with intact skin | |  |
| 16B3.00 | Spontaneous bruising | |  |
| SE4..11 | Leg bruise | |  |
| 16B..00 | Bruising symptom | |  |
| SE3..11 | Arm bruise | |  |
| R027.11 | [D]Spontaneous bruising | |  |
| 2I15.00 | O/E - bruising | |  |
| SE0..12 | Bruise of head | |  |
| SE43.11 | Toenail bruise | |  |
| SE2..11 | Bruise, trunk | |  |
| SE1..11 | Bruise of eye | |  |
| SE30011 | Shoulder bruise | |  |
| 16B2.00 | Bruises easily | |  |
| SE0..11 | Bruise of face | |  |
| 16B4.00 | Post-traumatic bruising | |  |
| 16BZ.00 | Bruising symptom NOS | |  |
| SC13.11 | Late effect of bruising | |  |
| SE42011 | Heel bruise | |  |
| SE06.00 | Bruise of mandibular joint area | |  |
| SE24211 | Bruise of scrotum | |  |
| SE05.11 | Bruise of ear | |  |
| SE11.11 | Bruise of eyelids | |  |
| SE11.12 | Bruise of periocular tissue | |  |
| SE05.12 | Bruise of auricle | |  |
| L336.11 | Bruising of cord | |  |
| Q416100 | Perinatal cutaneous bruising | |  |
|  |  | |  |
| **Cervical lymphadenopathy** | | |  |
| R056000 | [D]Lymphadenopathy | |  |
| 2C32.00 | O/E -cervical lymphadenopathy | |  |
| 2C3..00 | O/E - lymphadenopathy | |  |
| M043.00 | Acute lymphadenitis of face, head and neck | |  |
| 7N89000 | [SO]Cervical lymph node | |  |
| R056400 | [D]Localized enlarged lymph nodes | |  |
| 2C38.00 | O/E - post-auricular lymphadenopathy | |  |
| 2C3..12 | O/E - enlarged lymph nodes | |  |
| 2C46.00 | O/E - lymph nodes tender | |  |
| 2C3Z.00 | O/E - lymphadenopathy NOS | |  |
| 7N8C.00 | [SO]Other lymph node | |  |
| 2C41.00 | O/E - lymph nodes firm | |  |
|  |  | |  |
| **Change in bowel habit** | | |  |
| 19EA.00 | Change in bowel habit | |  |
| 19EA.11 | Altered bowel habit | |  |
| R078.00 | [D]Change in bowel habit | |  |
|  |  | |  |
| **Chest pain** |  | |  |
| 182..00 | Chest pain | |  |
| R065A00 | [D]Musculoskeletal chest pain | |  |
| 1828.00 | Atypical chest pain | |  |
| R065200 | [D]Anterior chest wall pain | |  |
| R065.00 | [D]Chest pain | |  |
| 1D22.11 | C/O - a chest wall symptom | |  |
| R065z00 | [D]Chest pain NOS | |  |
| R065600 | [D]Chest discomfort | |  |
| 1824.00 | Anterior chest wall pain | |  |
| 182C.00 | Chest wall pain | |  |
| 182Z.00 | Chest pain NOS | |  |
| 1822.00 | Central chest pain | |  |
| 182A.00 | Chest pain on exertion | |  |
| R065B00 | [D]Non cardiac chest pain | |  |
| 182B000 | Costal margin chest pain | |  |
| R065000 | [D]Chest pain, unspecified | |  |
| 1D22000 | Chest wall tenderness | |  |
| R065B14 | [D]Non-cardiac chest pain | |  |
| 1D22.00 | Symptom: chest wall | |  |
| R065C00 | [D]Retrosternal chest pain | |  |
| R065900 | [D]Parasternal chest pain | |  |
| R065D00 | [D]Central chest pain | |  |
| Ryu0400 | [X]Other chest pain | |  |
| R065011 | [D] Retrosternal chest pain | |  |
|  |  | |  |
| **Constipation** |  | |  |
| 19C..00 | Constipation | |  |
| 19C..11 | Constipation symptom | |  |
| J520z00 | Constipation NOS | |  |
| J520.00 | Constipation - functional | |  |
| 19C2.00 | Constipated | |  |
| 19CZ.00 | Constipation NOS | |  |
| 19EC.00 | Painful defaecation | |  |
| J520100 | Chronic constipation with overflow | |  |
| J520000 | Acute constipation | |  |
| J520200 | Chronic constipation without overflow | |  |
| J520300 | Drug induced constipation | |  |
| R079.00 | [D] Defaecation painful | |  |
| E264500 | Psychogenic constipation | |  |
| J520y00 | Other specified constipation | |  |
| J52y100 | Difficulty in ability to defaecate | |  |
| 2AF2.00 | O/E - defaec.ref.abn.-constip. | |  |
|  |  | |  |
| **Cough** |  | |  |
| 171..00 | Cough | |  |
| 171..11 | C/O - cough | |  |
| 1717.00 | Night cough present | |  |
| 1719.00 | Chesty cough | |  |
| R062.00 | [D]Cough | |  |
| 1718.00 | Night cough absent | |  |
| 1712.00 | Dry cough | |  |
| 173B.00 | Nocturnal cough / wheeze | |  |
| 1716.00 | Productive cough NOS | |  |
| 1714.00 | Productive cough -green sputum | |  |
| 171B.00 | Persistent cough | |  |
| 1715.00 | Productive cough-yellow sputum | |  |
| A33..00 | Whooping cough | |  |
| 1713.00 | Productive cough -clear sputum | |  |
| 171Z.00 | Cough symptom NOS | |  |
| 1716.11 | Coughing up phlegm | |  |
| 1719.11 | Bronchial cough | |  |
| 171A.00 | Chronic cough | |  |
| 174..11 | Hiccough symptom | |  |
| R068.00 | [D]Hiccough | |  |
| 171C.00 | Morning cough | |  |
| H310100 | Smokers' cough | |  |
| 171F.00 | Cough with fever | |  |
| 1419.11 | H/O: whooping cough | |  |
| R062000 | [D]Cough syncope | |  |
| R063000 | [D]Cough with haemorrhage | |  |
| 171J.00 | Reflux cough | |  |
| A33z.00 | Whooping cough NOS | |  |
| 65VA.00 | Notification of whooping cough | |  |
| 171D.00 | Evening cough | |  |
| 171H.00 | Difficulty in coughing up sputum | |  |
| S120A00 | Cough fracture | |  |
| S127100 | Cough fracture of ribs | |  |
| E261100 | Psychogenic cough | |  |
| 174..00 | Hiccough | |  |
| 655..11 | Whooping cough vaccination | |  |
| 25P5.00 | O/E-hernia-cough impulse shown | |  |
| 171E.00 | Unexplained cough | |  |
| 1742.00 | Hiccough present | |  |
| H243.00 | Pneumonia with whooping cough | |  |
| Eu45316 | [X]Psychogenic cough | |  |
| 1D87.00 | Cough aggravates symptom | |  |
| 171G.00 | Bovine cough | |  |
| ZV03611 | [V]Whooping cough vaccination | |  |
| ZV74B00 | [V]Screening for whooping cough | |  |
| 171K.00 | Barking cough | |  |
| 174Z.00 | Hiccough NOS | |  |
| A33y.00 | Whooping cough - other specified organism | |  |
| A33yz00 | Other whooping cough NOS | |  |
| E261200 | Psychogenic hiccough | |  |
| Eu45321 | [X]Psychogenic hiccough | |  |
|  |  | |  |
| **Diarrhoea** |  | |  |
| 19F..11 | Diarrhoea | |  |
| 19F..00 | Diarrhoea symptoms | |  |
| 19FZ.11 | Diarrhoea & vomiting, symptom | |  |
| A083.11 | Diarrhoea & vomiting -? infect | |  |
| 19G..00 | Diarrhoea and vomiting | |  |
| 19F2.00 | Diarrhoea | |  |
| 19F..12 | Loose stools | |  |
| A083.00 | Diarrhoea of presumed infectious origin | |  |
| 19FZ.00 | Diarrhoea symptom NOS | |  |
| J43z.11 | Chronic diarrhoea | |  |
| A076.11 | Viral diarrhoea | |  |
| A082.00 | Infectious diarrhoea | |  |
| J525.00 | Functional diarrhoea | |  |
| J4...13 | Noninfective diarrhoea | |  |
| A082000 | Dysenteric diarrhoea | |  |
| R077100 | [D] Stools loose | |  |
| A082z00 | Infectious diarrhoea NOS | |  |
| 19EE.00 | Increased frequency of defaecation | |  |
| E264300 | Psychogenic diarrhoea | |  |
| J521000 | Irritable bowel syndrome with diarrhoea | |  |
| J4zz.11 | Diarrhoea - presumed non-infectious | |  |
| E264311 | Spurious diarrhoea | |  |
| J4z..11 | Presumed noninfectious diarrhoea | |  |
| J432.11 | Allergic diarrhoea | |  |
| 19F3.00 | Spurious (overflow) diarrhoea | |  |
| J433.11 | Dietetic diarrhoea | |  |
| Eu45317 | [X]Psychogenic diarrhoea | |  |
| Ayu0H00 | [X]Diarrhoea+gastroenteritis of presumed infectious origin | |  |
|  |  | |  |
| **Dyspepsia** |  | |  |
| 1954.00 | Indigestion | |  |
| J16y400 | Dyspepsia | |  |
| 1955.00 | Heartburn | |  |
| E264400 | Psychogenic dyspepsia | |  |
| R071.00 | [D]Heartburn | |  |
| 195..00 | Indigestion symptoms | |  |
| 1955.11 | Heartburn symptom | |  |
| 1DC1.00 | Burning pain | |  |
| J16y412 | Indigestion NOS | |  |
| J16y411 | Flatulent dyspepsia | |  |
| Eu45318 | [X]Psychogenic dyspepsia | |  |
| R071z00 | [D]Heartburn NOS | |  |
| 195Z.00 | Indigestion symptom NOS | |  |
| 1958.00 | Undiagnosed dyspepsia | |  |
| SH72.00 | Burn of the oesophagus | |  |
| 8Hl0.00 | Referral to dyspepsia specialist nurse | |  |
|  |  | |  |
| **Dysphagia** |  | |  |
| R072000 | [D]Difficulty in swallowing | |  |
| 194..11 | Dysphagia | |  |
| R072.00 | [D]Dysphagia | |  |
| 1942.00 | Difficulty swallowing solids | |  |
| 194..00 | Swallowing symptoms | |  |
| ZV41612 | [V]Problems with swallowing | |  |
| 1944.00 | Painful swallowing | |  |
| 8B39400 | Drug not taken - prob.swallow. | |  |
| 1943.00 | Difficulty swallowing liquids | |  |
| R072z00 | [D]Dysphagia NOS | |  |
| 8B3O300 | Drug declined by patient - problem swallowing | |  |
| 194Z.00 | Swallowing symptom NOS | |  |
| 1944.11 | Odynophagia | |  |
| D00y000 | Sideropenic dysphagia | |  |
| ZV41600 | [V]Problem with swallowing or mastication | |  |
|  |  | |  |
| **Dyspnoea** |  | |  |
| 1739.00 | Shortness of breath | |  |
| R060800 | [D]Shortness of breath | |  |
| 173..00 | Breathlessness | |  |
| 173..13 | Shortness of breath symptom | |  |
| R060A00 | [D]Dyspnoea | |  |
| 173..11 | Breathlessness symptom | |  |
| 173..12 | Dyspnoea - symptom | |  |
| 173C.00 | Short of breath on exertion | |  |
| 1738.00 | Difficulty breathing | |  |
| 1732.00 | Breathless - moderate exertion | |  |
| 1733.00 | Breathless - mild exertion | |  |
| 1736.00 | Paroxysmal nocturnal dyspnoea | |  |
| R060D00 | [D]Breathlessness | |  |
| 2322.00 | O/E - dyspnoea | |  |
| 1734.00 | Breathless - at rest | |  |
| 1735.00 | Breathless - lying flat | |  |
| 173Z.00 | Breathlessness NOS | |  |
| 173D.00 | Nocturnal dyspnoea | |  |
| 173G.00 | Breathless - strenuous exertion | |  |
| 232B.00 | O/E - air hunger | |  |
| 173C.11 | Dyspnoea on exertion | |  |
| E261000 | Psychogenic air hunger | |  |
| 173F.00 | Short of breath dressing/undressing | |  |
|  |  | |  |
| **Haematuria** |  | |  |
| K197.00 | Haematuria | |  |
| 14D5.00 | H/O: haematuria | |  |
| 1A45.00 | Blood in urine - haematuria | |  |
| 1A45.12 | Haematuria - symptom | |  |
| 1A45.11 | Blood in urine - symptom | |  |
| K197300 | Frank haematuria | |  |
| K197000 | Painless haematuria | |  |
| K0A2.00 | Recurrent and persistent haematuria | |  |
| K197100 | Painful haematuria | |  |
| K197.11 | Traumatic haematuria | |  |
| K032100 | Recurrent benign haematuria syndrome | |  |
| K197400 | Clot haematuria | |  |
| K197.12 | Essential haematuria | |  |
| 4625.00 | Urine: red - blood | |  |
| K0A2600 | Recurrent and persistent haematuria, dense deposit disease | |  |
|  |  | |  |
| **Haemoptysis** |  | |  |
| R063.00 | [D]Haemoptysis | |  |
| 172..12 | Haemoptysis - symptom | |  |
| 172..00 | Blood in sputum - haemoptysis | |  |
| 172..11 | Blood in sputum - symptom | |  |
| R063z00 | [D]Haemoptysis NOS | |  |
| 4E35.00 | Sputum: blood cells present | |  |
| 4E24.00 | Sputum: contains blood | |  |
|  |  | |  |
| **Hoarseness** |  | |  |
| 1CA2.11 | Voice hoarseness | |  |
| 1CA2.00 | Hoarse | |  |
| 1CA..00 | Hoarseness symptom | |  |
| R044500 | [D]Hoarseness | |  |
| 1CA..11 | Hoarseness - throat symptom | |  |
| 2DE4.00 | O/E - hoarseness | |  |
| 1CAZ.00 | Hoarseness symptom NOS | |  |
|  |  | |  |
| **Intermenstrual bleeding** | | |  |
| K595.11 | Intermenstrual bleeding - regular | |  |
| K59y300 | Intermenstrual bleeding | |  |
| K596.11 | Intermenstrual bleeding - irregular | |  |
| 1582.00 | H/O: inter-menstrual bleeding | |  |
|  |  | |  |
| **Jaundice** |  | |  |
| R024.00 | [D]Jaundice (not of newborn) | |  |
| R024111 | [D]Jaundice | |  |
| 1675.11 | Jaundice - symptom | |  |
| J66y600 | Obstructive jaundice NOS | |  |
| 14C6.00 | H/O: jaundice | |  |
| 2274.11 | O/E - jaundiced | |  |
| 1675.00 | Yellow/jaundiced colour | |  |
| 1675.12 | Yellow - symptom | |  |
| 2274.00 | O/E - jaundiced colour | |  |
| A60..00 | Yellow fever | |  |
| 65V3.00 | Notification of inf. jaundice | |  |
| R024z00 | [D]Jaundice (not of newborn) NOS | |  |
| A60z.00 | Yellow fever unspecified | |  |
| D100.11 | Acholuric familial jaundice | |  |
| AA00.11 | Spirochaetal jaundice | |  |
|  |  | |  |
| **Loin pain** |  | |  |
| 1A53.11 | C/O - loin pain | |  |
| R090C00 | [D]Loin pain | |  |
|  |  | |  |
| **Lump in head and neck** | | |  |
| R022200 | [D]Lump, localized and superficial | |  |
| 2I11.00 | O/E - a lump | |  |
| R042200 | [D]Lump in head or neck | |  |
| R022.00 | [D]Local superficial swelling, mass or lump | |  |
| 2I1A.00 | Lump on neck | |  |
| 1CB4.00 | Feeling of lump in throat | |  |
| R042500 | [D]Localized swelling, mass and lump, neck | |  |
| R042z11 | [D]Lump throat | |  |
| R042.00 | [D]Swelling, mass or lump in head and neck | |  |
| R042800 | [D]Lump on head | |  |
| R022800 | [D]Lump on back | |  |
| 22G5.00 | Parotid lump | |  |
| R042.12 | [D]Swelling, mass or lump in neck | |  |
| R042A00 | [D]Submental lump | |  |
| 22H4.00 | O/E - thyroid lump | |  |
| R042400 | [D]Localized swelling, mass and lump, head | |  |
| R042900 | [D]Lump on nose | |  |
| R022z00 | [D]Local superficial swelling, mass or lump NOS | |  |
| R042.11 | [D]Swelling, mass or lump in head | |  |
| R042z00 | [D]Swelling, mass or lump in head or neck NOS | |  |
|  |  | |  |
| **Lump** |  | |  |
| R022200 | [D]Lump, localized and superficial | |  |
| 2I11.00 | O/E - a lump | |  |
| R042200 | [D]Lump in head or neck | |  |
| R022.00 | [D]Local superficial swelling, mass or lump | |  |
| 2I1A.00 | Lump on neck | |  |
| R093B00 | [D]Groin lump | |  |
| 1CB4.00 | Feeling of lump in throat | |  |
| R022700 | [D]Axillary lump | |  |
| R042500 | [D]Localized swelling, mass and lump, neck | |  |
| R066100 | [D]Chest lump | |  |
| R022900 | [D]Foot lump | |  |
| R093200 | [D]Abdominal lump | |  |
| R042z11 | [D]Lump throat | |  |
| R022400 | [D]Localized swelling, mass and lump, upper limb | |  |
| R022B00 | [D]Lump on hand | |  |
| R022D00 | [D]Lump on leg | |  |
| R093.00 | [D]Swelling, mass or lump within abdomen or pelvis | |  |
| R022G00 | [D]Finger lump | |  |
| R042.00 | [D]Swelling, mass or lump in head and neck | |  |
| R022500 | [D]Localized swelling, mass and lump, lower limb | |  |
| R022H00 | [D]Wrist lump | |  |
| R042800 | [D]Lump on head | |  |
| R022800 | [D]Lump on back | |  |
| R022I00 | [D]Toe lump | |  |
| R022C00 | [D]Lump on knee | |  |
| 22G5.00 | Parotid lump | |  |
| R042.12 | [D]Swelling, mass or lump in neck | |  |
| R022F00 | [D]Lump on thigh | |  |
| R022E00 | [D]Lump on shin | |  |
| R022A00 | [D]Shoulder lump | |  |
| R042A00 | [D]Submental lump | |  |
| 22H4.00 | O/E - thyroid lump | |  |
| R066.00 | [D]Swelling, mass and lump of chest | |  |
| R042400 | [D]Localized swelling, mass and lump, head | |  |
| R042900 | [D]Lump on nose | |  |
| R093800 | [D]Umbilical lump | |  |
| R093500 | [D]Pelvic lump | |  |
| R022z00 | [D]Local superficial swelling, mass or lump NOS | |  |
| R042.11 | [D]Swelling, mass or lump in head | |  |
| R022600 | [D]Localized swelling, mass and lump, multiple sites | |  |
| R042z00 | [D]Swelling, mass or lump in head or neck NOS | |  |
| R066z00 | [D]Swelling, mass or lump of chest NOS | |  |
| R093111 | [D]Lump stomach | |  |
|  |  | |  |
| **Lower urinary tract symptoms** | | |  |
| 1A...12 | Urinary symptoms | |  |
| 1A...00 | Genitourinary symptoms | |  |
| R08..00 | [D]Urinary system symptoms | |  |
| 1A4..11 | Urine appearance symptom | |  |
| R08z.00 | [D]Other urinary system symptom | |  |
| 1AZZ.00 | Genitourinary symptom NOS | |  |
| 1AZ..00 | Genitourinary symptoms NOS | |  |
| R08zz00 | [D]Urinary system symptoms NOS | |  |
| E265.00 | Psychogenic genitourinary tract symptoms | |  |
| 66K3.00 | Urinary symptom change | |  |
| E265z00 | Psychogenic genitourinary tract symptom NOS | |  |
| Ryu4.00 | [X]Symptoms and signs involving the urinary system | |  |
|  |  | |  |
| **Microscopic haematuria** | | |  |
| K197200 | Microscopic haematuria | |  |
| 4695.00 | Urine blood test = + | |  |
| 4697.00 | Urine blood test = +++ | |  |
| 4696.00 | Urine blood test = ++ | |  |
| 4693.00 | Urine: trace non-haemol. blood | |  |
| 46G2.11 | RBCs- red blood cells in urine | |  |
| 4694.00 | Urine: trace haemolysed blood | |  |
| K0A2700 | Recur+persist haematuria difus crescentic glomerulonephritis | |  |
| K0A2200 | Recur+persist haematuria difus membranous glomerulonephritis | |  |
| K0A2000 | Recurrent+persistnt haematuria minor glomerular abnormality | |  |
| K0A2600 | Recurrent and persistent haematuria, dense deposit disease | |  |
| K0A2100 | Recur+persist haematuria, focal+segmental glomerular lesions | |  |
|  |  | |  |
| **Night sweats** |  | |  |
| 1662.00 | Excessive sweating | |  |
| 166..00 | Sweating symptom | |  |
| 1662.12 | Night sweats | |  |
| R008100 | [D]Excessive sweating | |  |
| 2223.00 | O/E - sweating | |  |
| 166Z.00 | Sweating symptom NOS | |  |
| R008400 | [D]Night sweats | |  |
|  |  | |  |
| **Nipple discharge** |  | |  |
| 26C3.11 | Sore nipple | |  |
| K317400 | Nipple discharge | |  |
| 1A9..00 | Nipple discharge symptom | |  |
| K317300 | Inversion of nipple | |  |
| 26C3.12 | Painful nipple | |  |
| K317500 | Retraction of nipple | |  |
| K312.11 | Cracked nipple | |  |
| 26D5.00 | O/E - nipple disch.-blood-dark | |  |
| K312.00 | Fissure of nipple | |  |
| 26C3.00 | O/E - cracked nipple | |  |
| 26D..00 | O/E - nipple discharge | |  |
| 7136500 | Eversion of nipple | |  |
| 1A92.00 | Nipple discharge present | |  |
| 26C2.11 | O/E - retracted nipple | |  |
| 1A9Z.00 | Nipple discharge NOS | |  |
| 26E2.00 | O/E-breast lump-nipple/central | |  |
| 26C2.00 | O/E - retraction of nipple | |  |
| 7136300 | Reconstruction of the nipple or areolar complex unspecified | |  |
| 26D4.00 | O/E-nipple discharge-blood-red | |  |
| 26D3.00 | O/E - nipple discharge - milky | |  |
| 26DZ.00 | O/E - nipple discharge NOS | |  |
| 26D1.00 | O/E - no nipple discharge | |  |
| 26D2.00 | O/E - nipple discharge - clear | |  |
| 26D6.00 | O/E - nipple discharge - pus | |  |
|  |  | |  |
| **Pain in testis** |  | |  |
| R090B00 | [D]Groin pain | |  |
| K28z.11 | Pain in testis | |  |
| K28y811 | Testicular pain | |  |
| K28y800 | Pain in testis | |  |
| 1A5C.00 | Pain in scrotum | |  |
| 1A57.00 | Pain in testicle | |  |
| 1A57.11 | Testicular pain | |  |
|  |  | |  |
| **Pelvic pain** |  | |  |
| 1A59.00 | C/O pelvic pain | |  |
| 1979.00 | Suprapubic pain | |  |
| K583.11 | Painful menorrhoea | |  |
| R090G11 | [D] Pelvic pain | |  |
| R090G00 | [D]Pelvic and perineal pain | |  |
| 1574.11 | H/O: painful periods | |  |
| 1A58.00 | Pain in female genitalia | |  |
| 1A5A.00 | C/O perineal pain | |  |
| N33A000 | Bony pelvic pain | |  |
| K58y000 | Other pelvic pain - female | |  |
|  |  | |  |
| **Postcoital bleeding** | |  | |
| K597.00 | Postcoital bleeding | |  |
| 1581.00 | H/O: post-coital bleeding | |  |
| K587.00 | Contact bleeding of cervix | |  |
| K59B.00 | Postmenopausal postcoital bleeding | |  |
| K59A.00 | Premenopausal postcoital bleeding | |  |
|  |  | |  |
| **Postmenopausal bleeding** | | |  |
| K5A1.00 | Postmenopausal bleeding | |  |
| 1583.00 | H/O: post-menopausal bleeding | |  |
|  |  | |  |
| **Pruritis** |  | |  |
| M18z.00 | Pruritus NOS | |  |
| M180.00 | Pruritus ani | |  |
| M181.11 | Pruritus vulvae | |  |
| M18..00 | Pruritus and related conditions | |  |
| M18y200 | Pruritus senilis | |  |
| M181.00 | Pruritus of genital organs | |  |
| M181100 | Pruritus vulvae | |  |
| Eu45y13 | [X]Psychogenic pruritis | |  |
| M181000 | Pruritus scroti | |  |
| E263000 | Psychogenic pruritus | |  |
| Myu2D00 | [X]Pruritus, unspecified | |  |
| M18yz11 | Aquagenic pruritis | |  |
| M18yz00 | Other pruritic conditions NOS | |  |
| M18y.00 | Other specified pruritic conditions | |  |
| M18y100 | Pruritus hiemalis | |  |
| Myu2B00 | [X]Other pruritus | |  |
| M18y300 | Brachioradial pruritis | |  |
| M18y000 | Pruritus aestivalis | |  |
|  |  | |  |
| **Sore throat** |  | |  |
| 1C9..00 | Sore throat symptom | |  |
| 1C9..11 | Throat soreness | |  |
| 1922.00 | Sore mouth | |  |
| H02..11 | Sore throat NOS | |  |
| 2DC3.00 | Inflamed throat | |  |
| 1C92.00 | Has a sore throat | |  |
| 1CB3.00 | Throat pain | |  |
| 1CB..00 | Throat symptom NOS | |  |
| A340.00 | Streptococcal sore throat | |  |
| H02..12 | Viral sore throat NOS | |  |
| H03..11 | Throat infection - tonsillitis | |  |
| 1923.11 | Sore gums - symptom | |  |
| 1922.11 | Sore mouth - symptom | |  |
| 1CB5.00 | Throat irritation | |  |
| 1C9Z.00 | Sore throat symptom NOS | |  |
| 1923.00 | Sore gums | |  |
| 1CBZ.00 | Throat symptom NOS | |  |
| H121.11 | Sore throat - chronic | |  |
| R041.00 | [D]Throat pain | |  |
| 1C93.00 | Persistent sore throat | |  |
| A340z00 | Streptococcal sore throat NOS | |  |
| A34z.00 | Streptococcal sore throat with scarlatina NOS | |  |
| A34..00 | Streptococcal sore throat and scarlatina | |  |
|  |  | |  |
| **Stomach fullness** |  | |  |
| 1984.00 | Upset stomach | |  |
| 7N30100 | [SO]Stomach | |  |
| J16z.00 | Stomach function disorder NOS | |  |
| J16..00 | Disorders of stomach function | |  |
| J1...00 | Oesophageal, stomach and duodenal diseases | |  |
| J16y.00 | Other specified stomach function disorders | |  |
|  |  | |  |
| **Stridor** |  | |  |
| 1737.00 | Wheezing | |  |
| 1737.11 | Wheezing symptom | |  |
| 173B.00 | Nocturnal cough / wheeze | |  |
| 2326.00 | O/E - expiratory wheeze | |  |
| R060900 | [D]Wheezing | |  |
| R061.00 | [D]Stridor | |  |
| 2DE2.00 | O/E - stridor present | |  |
| P83y900 | Congenital laryngeal stridor | |  |
| H1y7C11 | Laryngismus stridulus | |  |
|  |  | |  |
| **Testicular lump** |  | |  |
| K28yu11 | Testicular swelling NOS | |  |
| R093B00 | [D]Groin lump | |  |
| 2659.00 | Testicular lump | |  |
| 265..00 | O/E - scrotal swelling | |  |
| 2654.00 | O/E - testicular swelling | |  |
| R093900 | [D]Groin swelling | |  |
| 265..12 | O/E - testicular swelling | |  |
| R08z300 | [D]Swelling of scrotum | |  |
| 2652.00 | O/E -scrotal swelling-transill | |  |
| 265Z.00 | O/E - scrotal swelling NOS | |  |
| 265E.00 | O/E - left scrotal swelling | |  |
| 265D.00 | O/E - right scrotal swelling | |  |
| 2653.00 | O/E -scrotal swell.no transill | |  |
|  |  | |  |
| **Thrombocytosis** |  | |  |
| 42P3.00 | Thrombocythaemia | |  |
| D3y0.00 | Essential thrombocytosis | |  |
| B937400 | Essential (haemorrhagic) thrombocythaemia | |  |
| B937500 | Idiopathic thrombocythaemia | |  |
| B937411 | Primary thrombocythaemia | |  |
| BBs4.00 | [M]Idiopathic thrombocythaemia | |  |
| B937.12 | Idiopathic thrombocythaemia | |  |
|  |  | |  |
| **Ulceration** |  | |  |
| J082.11 | Mouth ulcer | |  |
| M27z.00 | Chronic skin ulcer NOS | |  |
| 2FF..00 | O/E - skin ulcer | |  |
| B33..13 | Rodent ulcer | |  |
| M27..00 | Chronic skin ulcer | |  |
| M272.00 | Ulcer of skin | |  |
| 2533.11 | O/E - mouth ulcer | |  |
| 2567.00 | O/E - ulcer on tongue | |  |
| 14F3.00 | H/O: chronic skin ulcer | |  |
| 2FF2.00 | O/E - skin ulcer present | |  |
| J085800 | Lip ulcer | |  |
| 2533.00 | O/E - mouth ulcer present | |  |
| M07z.12 | Infected skin ulcer | |  |
| J090100 | Traumatic ulceration of tongue | |  |
| F4A0z00 | Corneal ulcer NOS | |  |
| H022.00 | Acute ulcerative pharyngitis | |  |
| H1y1200 | Nasal septum ulcer | |  |
| J08z800 | Traumatic ulcer of oral mucosa | |  |
| J082211 | Recurrent mouth ulcers | |  |
| 1485.00 | H/O: corneal ulcer | |  |
| F4A0100 | Marginal corneal ulcer | |  |
| S8z..12 | Traumatic ulcer NOS | |  |
| H14y400 | Tonsil ulcer | |  |
| F503300 | Ulcer of pinna | |  |
| F4Dy000 | Ulcer of eyelid | |  |
| 2FFZ.00 | O/E - skin ulcer NOS | |  |
| J082100 | Major aphthous ulceration | |  |
| J14..14 | Marginal ulcer | |  |
| J082300 | Herpetic aphthous ulceration | |  |
| H5y1400 | Ulcer of trachea | |  |
|  |  | |  |
| **Unspecified uterine and bleeding bleeding** | | |  |
| K5E..00 | Other abnormal uterine and vaginal bleeding | |  |
| K5E2.00 | Abnormal vaginal bleeding, unspecified | |  |
| K5Ez.00 | Abnormal uterine and vaginal bleeding, unspecified | |  |
| K5E1.00 | Abnormal uterine bleeding, unspecified | |  |
| 158Z.00 | H/O:abnormal uterine bleed NOS | |  |
| K5E0.00 | Abnormal uterine bleeding unrelated to menstrual cycle | |  |
| Kyu9D00 | [X]Other specified abnormal uterine and vaginal bleeding | |  |
|  |  | |  |
| **Urinary tract infections** | | |  |
| K190z00 | Urinary tract infection, site not specified NOS | |  |
| K190.00 | Urinary tract infection, site not specified | |  |
| 1J4..00 | Suspected UTI | |  |
| 1AG..00 | Recurrent urinary tract infections | |  |
| K190311 | Recurrent UTI | |  |
| K190.11 | Recurrent urinary tract infection | |  |
| K190300 | Recurrent urinary tract infection | |  |
| A78A000 | Chlamydial infection of lower genitourinary tract | |  |
| A78AX00 | Chlamydial infection of genitourinary tract, unspecified | |  |
| K190500 | Urinary tract infection | |  |
| K190400 | Chronic urinary tract infection | |  |
| Ayu4K00 | [X]Chlamydial infection of genitourinary tract, unspecified | |  |
|  |  | |  |
| **Vaginal discharge** |  | |  |
| 1A7..00 | Vaginal discharge symptom | |  |
| 26AZ.00 | O/E - vaginal discharge NOS | |  |
| 26A..00 | O/E - vaginal discharge | |  |
| K565.12 | Vaginal discharge NOS | |  |
| 1A72.00 | Vaginal discharge present | |  |
| 1A7Z.00 | Vaginal discharge NOS | |  |
| 26A2.00 | O/E - white vag. discharge | |  |
| 26A3.00 | O/E - creamy vag. discharge | |  |
| 26A4.00 | O/E - yellow vag. discharge | |  |
| 26A9.00 | O/E - offensive vag. discharge | |  |
| 26A5.00 | O/E - green vag. discharge | |  |
| 26A7.00 | O/E - frothy vag. discharge | |  |
| 26A8.00 | O/E - profuse vag. discharge | |  |
|  |  | |  |
| **Vomiting** |  | |  |
| 1992.00 | Vomiting | |  |
| 19FZ.11 | Diarrhoea & vomiting, symptom | |  |
| 199..14 | Vomiting symptoms | |  |
| 199..00 | Vomiting | |  |
| R070100 | [D]Vomiting | |  |
| R070.00 | [D]Nausea and vomiting | |  |
| A076.12 | Viral vomiting | |  |
| 199..11 | C/O - vomiting | |  |
| J680.11 | Vomiting of blood | |  |
| 1993.00 | Projectile vomiting | |  |
| E264200 | Cyclical vomiting - psychogenic | |  |
| 4A24.11 | Coffee ground vomit | |  |
| R070z00 | [D]Nausea and vomiting NOS | |  |
| J162.00 | Persistent vomiting | |  |
| 199Z.00 | Vomiting NOS | |  |
| A78y111 | Winter vomiting disease | |  |
| J162000 | Cyclical vomiting NOS | |  |
| 1994.11 | Blood in vomit - symptom | |  |
| 1996.00 | Vomiting - bile stained | |  |
| E275400 | Psychogenic vomiting NOS | |  |
| R070300 | [D]Drug induced vomiting | |  |
| A78y100 | Epidemic vomiting syndrome | |  |
| Eu50511 | [X]Psychogenic vomiting | |  |
| 1994.00 | Vomiting blood - fresh | |  |
| 4A1..11 | Vomit - O/E, general | |  |
| 1995.00 | Vomiting blood - coffee ground | |  |
| J16y500 | Functional vomiting | |  |
| R070400 | [D]Projectile vomiting | |  |
| 4A...00 | Vomit examination | |  |
| 4A25.11 | Bilious vomit O/E | |  |
| Eu50500 | [X]Vomiting associated with other psychological disturbances | |  |
| 4A5..11 | Occult blood in vomit | |  |
| J162z00 | Persistent vomiting NOS | |  |
| 4A2..00 | Vomit appearance | |  |
| 4A26.11 | Faeculant vomit O/E | |  |
| 4A24.00 | Vomit: coffee ground | |  |
| 4A25.00 | Vomit: bilious | |  |
| 4A22.00 | Vomit appearance - abnormal | |  |
| 4A23.11 | Blood in vomit O/E | |  |
| 4A23.00 | Vomit: frank blood present | |  |
| 4A26.00 | Vomit: faeculant | |  |
| 4A5..00 | Vomit occult blood | |  |
| 4A4Z.00 | Vomit pH NOS | |  |
| 4A6..00 | Vomit toxicology | |  |
| 4A3..00 | Vomit odour | |  |
| 4A51.00 | Vomit occult blood positive | |  |
| 4A5Z.00 | Vomit occult blood NOS | |  |
| 4AZ..00 | Vomit examination NOS | |  |
| 4A1Z.00 | Vomit exam. general NOS | |  |
| 4A2Z.00 | Vomit: appearance NOS | |  |
| 4A27.00 | Vomit: mucous present | |  |
