# Supplementary material for: Age and Gender Variations in Cancer Diagnostic Intervals in 15 Cancers: Analysis of Data from the UK Clinical Practice Research Datalink
Source: PLoS One. 2015 May 15;10(5):e0127717. doi: 10.1371/journal.pone.0127717 (PMC4433335; doi:10.1371/journal.pone.0127717)
Supplement: S3 Table — (DOCX) [file pone.0127717.s003.docx]

**S3 Table. Cancer site non-specific symptom Read Codes**

**Symptoms are listed in an alphabetical order and used for all cancer sites**

| **Read code** | **Read term** |
| --- | --- |
| **Anaemia** |  |
| D21z.00 | Anaemia unspecified |
| D00..00 | Iron deficiency anaemias |
| D00y100 | Microcytic hypochromic anaemia |
| 145..11 | H/O: anaemia |
| D010.00 | Pernicious anaemia |
| D0...00 | Deficiency anaemias |
| D00zz00 | Iron deficiency anaemia NOS |
| 688..11 | Anaemia screen |
| L182500 | Iron deficiency anaemia of pregnancy |
| 2C2..11 | O/E - anaemic |
| D012500 | Macrocytic anaemia unspecified cause |
| D21..00 | Other and unspecified anaemias |
| D214.00 | Chronic anaemia |
| D21z.12 | Normocytic anaemia due to unspecified cause |
| Dyu0000 | [X]Other iron deficiency anaemias |
| D012.00 | Folate-deficiency anaemia |
| D011X00 | Vitamin B12 deficiency anaemia, unspecified |
| D00..12 | Microcytic - hypochromic anaemia |
| D00z.00 | Unspecified iron deficiency anaemia |
| D011.11 | Vitamin B12 deficiency anaemia |
| D0z..00 | Deficiency anaemias NOS |
| D1...00 | Haemolytic anaemias |
| D21z.11 | Secondary anaemia NOS |
| D20..00 | Aplastic anaemia |
| 1271 | FH: Anaemia |
| D000.00 | Iron deficiency anaemia due to chronic blood loss |
| 6884 | Anaemia screen |
| 2C2..00 | O/E - anaemia |
| D00..11 | Hypochromic - microcytic anaemia |
| D01..11 | Megaloblastic anaemia |
| D21z.13 | Macrocytic anaemia of unspecified cause |
| B937X00 | Refractory anaemia, unspecified |
| 688..00 | Anaemia/blood screening |
| 1451 | H/O: anaemia - iron deficient |
| D011.00 | Other vitamin B12 deficiency anaemias |
| D2z..00 | Other anaemias NOS |
| D210.00 | Sideroblastic anaemia |
| D001.00 | Iron deficiency anaemia due to dietary causes |
| D10z.00 | Hereditary haemolytic anaemia NOS |
| 1454 | H/O: anaemia NOS |
| D01z000 | [X]Megaloblastic anaemia NOS |
| D211.00 | Acute posthaemorrhagic anaemia |
| 1453 | H/O: haemolytic anaemia |
| Dyu2200 | [X]Anaemia in other chronic diseases classified elsewhere |
| D01..00 | Other deficiency anaemias |
| 688Z.00 | Anaemia/blood screen NOS |
| D212.00 | Anaemia in neoplastic disease |
| D00yz00 | Other specified iron deficiency anaemia NOS |
| 2C2Z.00 | O/E - anaemia NOS |
| D2y..00 | Other specified anaemias |
| D011z00 | Other vitamin B12 deficiency anaemia NOS |
| D012.11 | Folic acid deficiency anaemia |
| D000.11 | Normocytic anaemia due to chronic blood loss |
| D00y.00 | Other specified iron deficiency anaemia |
| D013000 | Combined B12 and folate deficiency anaemia |
| D1z..00 | Haemolytic anaemias NOS |
| 2C23.00 | O/E - clinically anaemic |
| D21yz00 | Other specified anaemia NOS |
| D00z200 | Idiopathic hypochromic anaemia |
| D013.00 | Other specified megaloblastic anaemia NEC |
| Dyu0200 | [X]Other vitamin B12 deficiency anaemias |
| B937000 | Refractory anaemia without sideroblasts, so stated |
| D011000 | Vitamin B12 deficiency anaemia due to dietary causes |
| D11..00 | Acquired haemolytic anaemias |
| B937100 | Refractory anaemia with sideroblasts |
| D21y.00 | Other specified anaemias |
| Dyu0600 | [X]Vitamin B12 deficiency anaemia, unspecified |
| D0y..00 | Other specified deficiency anaemias |
| D201.00 | Acquired aplastic anaemia |
| D000.12 | Iron deficiency anaemia due to blood loss |
| 1452 | H/O: Anaemia vit.B12 deficient |
| D2...00 | Aplastic and other anaemias |
| B937200 | Refractory anaemia with excess of blasts |
| D012z00 | Folate-deficiency anaemia NOS |
| D201000 | Aplastic anaemia due to chronic disease |
| D110000 | Primary cold-type haemolytic anaemia |
| D012100 | Folate-deficiency anaemia due to dietary causes |
| D111100 | Microangiopathic haemolytic anaemia |
| D213.00 | Refractory Anaemia |
| 2C24.00 | O/E - profoundly anaemic |
| D11z.00 | Acquired haemolytic anaemia NOS |
| D01z.00 | Other deficiency anaemias NOS |
| D10..00 | Hereditary haemolytic anaemias |
| D106z00 | Sickle-cell anaemia NOS |
| D014.00 | Protein-deficiency anaemia |
| D21yy00 | Other specified other anaemia |
| D013z00 | Other specified megaloblastic anaemia NEC NOS |
| D201z00 | Acquired aplastic anaemia NOS |
| D1y..00 | Other specified haemolytic anaemias |
| 2C22.00 | O/E - equivocally anaemic |
| D211.11 | Normocytic anaemia following acute bleed |
| D201.11 | Normocytic anaemia due to aplasia |
| D201700 | Transient hypoplastic anaemia |
| Dyu0100 | [X]Other dietary vitamin B12 deficiency anaemia |
| Dyu2400 | [X]Other specified anaemias |
| D204.00 | Idiopathic aplastic anaemia |
| D200.00 | Constitutional aplastic anaemia |
| D20z.00 | Aplastic anaemia NOS |
| D111000 | Mechanical haemolytic anaemia |
| D012112 | Megaloblastic anaemia due to dietary causes |
| D201211 | Hypoplastic anaemia due to infection |
| D210100 | Acquired sideroblastic anaemia |
| D012300 | Folate-deficiency anaemia due to malabsorption |
| D106500 | Sickle-cell anaemia with haemoglobin E disease |
| D111.00 | Non-autoimmune haemolytic anaemia |
| D01z.11 | Megaloblastic anaemia NOS |
| D200011 | Constitutional aplastic anaemia without malformation |
| Dyu0.00 | [X]Nutritional anaemias |
| ZV78100 | [V]Screening for other or unspecified deficiency anaemia |
| D102200 | Drug-induced enzyme deficiency anaemia |
| D201412 | Hypoplastic anaemia due to toxic cause |
| D111z00 | Non-autoimmune haemolytic anaemia NOS |
|  |  |
| **Anorexia** |  |
| R030000 | [D]Appetite loss |
| R030.00 | [D]Anorexia |
| 1612.11 | Anorexia symptom |
| 1615 | Reduced appetite |
| 161..00 | Appetite symptom |
| 1612 | Appetite loss - anorexia |
| 1612.12 | Loss of appetite - symptom |
| 161Z.00 | Appetite symptom NOS |
| E275600 | Non-organic loss of appetite |
| Eu50y12 | [X]Psychogenic loss of appetite |
| R030z00 | [D]Anorexia NOS |
|  |  |
| **Fatigue** |  |
| R007500 | [D]Tiredness |
| 168..00 | Tiredness symptom |
| 1683 | Tired all the time |
| 1682 | Fatigue |
| E205.12 | Tired all the time |
| 168..11 | Fatigue - symptom |
| F286.00 | Chronic fatigue syndrome |
| R007100 | [D]Fatigue |
| F286.11 | CFS - Chronic fatigue syndrome |
| 168Z.00 | Tiredness symptom NOS |
| Eu46011 | [X]Fatigue syndrome |
| R007z00 | [D]Malaise and fatigue NOS |
| R007.00 | [D]Malaise and fatigue |
| F286100 | Moderate chronic fatigue syndrome |
| F286000 | Mild chronic fatigue syndrome |
| F286200 | Severe chronic fatigue syndrome |
|  |  |
| **Weight loss** |  |
| 162..00 | Weight symptom |
| 1625.11 | Abnormal weight loss - symptom |
| 1623 | Weight decreasing |
| R032.00 | [D]Abnormal loss of weight |
| 22AZ.00 | O/E - weight NOS |
| 1625 | Abnormal weight loss |
| 22A6.00 | O/E - Underweight |
| 1D1A.00 | Complaining of weight loss |
| R034100 | [D]Failure to gain weight |
| 162Z.00 | Weight symptom NOS |
| R034800 | [D]Underweight |
| 22A8.00 | Weight loss from baseline weight |
