# Supplementary material for: Age and Gender Variations in Cancer Diagnostic Intervals in 15 Cancers: Analysis of Data from the UK Clinical Practice Research Datalink
Source: PLoS One. 2015 May 15;10(5):e0127717. doi: 10.1371/journal.pone.0127717 (PMC4433335; doi:10.1371/journal.pone.0127717)
Supplement: S4 Table — (DOCX) [file pone.0127717.s004.docx]

**S4 Table. List of symptoms by cancer site**

| **Cancer** | **Symptom** |
| --- | --- |
| **For all** | Weight loss |
|  | Anorexia |
|  | Fatigue |
|  | Anaemia (<12.0g, men) |
|  | Anaemia (<11.0g, women) |
| **Colorectal** | Constipation |
|  | Diarrhoea |
|  | Rectal bleeding |
|  | Abdominal pain |
|  | Change in bowel habit |
| **Lung** | Cough |
|  | Dyspnoea |
|  | Chest pain |
|  | Thrombocytosis |
|  | Haemoptysis |
| **Breast** | Breast lump |
|  | Unilateral nipple eczema |
|  | Breast skin changes (peau d’orange) |
|  | Breast pain |
|  | Nipple discharge/bleeding |
| **Pancreas** | Painless jaundice |
|  | Abdominal/epigastric pain |
| **Oesophageal** | Dyspepsia |
|  | Vomiting |
|  | Pain swallowing (odynophagia) |
|  | Dysphagia |
| **Stomach** | Dyspepsia |
|  | Vomiting |
|  | Early satiation/fullness |
| **Uterus** | Inter-menstrual bleeding |
|  | Post-coital bleeding |
|  | Post-menopausal bleeding |
|  | Vaginal discharge |
|  | Pelvic pain |
| **Cervix** | Inter-menstrual bleeding |
|  | Post-coital bleeding |
|  | Post-menopausal bleeding |
|  | Vaginal discharge |
|  | Pelvic pain |
| **Kidney** | Macrocytic haematuria |
|  | Microcytic haematuria |
|  | Loin pain |
| **Bladder** | Macroscopic haematuria |
|  | Microscopic haematuria |
|  | UTIs |
|  | LUTS |
| **Testis** | Painless lump/swelling in testis |
|  | Pain in testis |
| **Oral/pharynx/larynx** | Sore throat |
|  | Stridor |
|  | Hoarseness |
|  | Dysphagia |
|  | Ulceration |
|  | Lump |
|  | Cervical lymphadenopathy |
| **Lymphoma** | Lump(s) |
|  | Night sweats |
|  | Bruising |
|  | Bleeding |
|  | Pruritus |
| **Leukaemia** | Bruising |
|  | Bleeding |
| **Myeloma** | Bone pain |
|  | Bruising |
|  | Bleeding |
