# Supplementary material for: Age and Gender Variations in Cancer Diagnostic Intervals in 15 Cancers: Analysis of Data from the UK Clinical Practice Research Datalink
Source: PLoS One. 2015 May 15;10(5):e0127717. doi: 10.1371/journal.pone.0127717 (PMC4433335; doi:10.1371/journal.pone.0127717)
Supplement: S5 Table — (DOCX) [file pone.0127717.s005.docx]

**S5 Table. List of NICE symptom categories by cancer site**

| **Cancer sites** | **Nice qualifying Symptoms** | **NICE non-qualifying symptoms** |
| --- | --- | --- |
| **Colorectal** | Change in bowel habit | Abdominal pain |
|  | Diarrhoea | Constipation |
|  | Rectal bleeding | Anorexia |
|  | Anaemia | Fatigue |
|  |  | Weight loss |
| **Lung** | Chest pain | Thrombocytosis |
|  | Cough | Anaemia |
|  | Dyspnoea | Anorexia |
|  | Haemoptysis | Fatigue |
|  | Weight loss |  |
| **Breast** | Breast lump | Breast pain |
|  | Breast skin changes (peau d’orange) | Anaemia |
|  | Nipple discharge/bleeding | Anorexia |
|  | Unilateral nipple eczema | Fatigue |
|  |  | Weight loss |
| **Pancreas** | Abdominal/epigastric pain | Anorexia |
|  | Painless jaundice | Fatigue |
|  | Anaemia |  |
|  | Weight loss |  |
| **Oesophageal** | Dyspepsia | Pain swallowing (odynophagia) |
|  | Dysphagia | Anorexia |
|  | Vomiting | Fatigue |
|  | Anaemia |  |
|  | Weight loss |  |
| **Stomach** | Dyspepsia | Early satiation/fullness |
|  | Vomiting | Anorexia |
|  | Anaemia | Fatigue |
|  | Weight loss |  |
| **Endometrial** | Inter-menstrual bleeding | Pelvic pain |
|  | Post-menopausal bleeding | Post-coital bleeding |
|  |  | Vaginal discharge |
|  |  | Anaemia |
|  |  | Anorexia |
|  |  | Fatigue |
|  |  | Weight loss |
| **Cervical** | Inter-menstrual bleeding | Pelvic pain |
|  | Post-menopausal bleeding | Post-coital bleeding |
|  |  | Vaginal discharge |
|  |  | Anaemia |
|  |  | Anorexia |
|  |  | Fatigue |
|  |  | Weight loss |
| **Kidney** | Macrocytic haematuria | Loin pain |
|  | Microcytic haematuria | Anaemia |
|  |  | Anorexia |
|  |  | Fatigue |
|  |  | Weight loss |
| **Bladder** | Macroscopic haematuria | LUTS |
|  | Microscopic haematuria | Anaemia |
|  | UTIs | Anorexia |
|  |  | Fatigue |
|  |  | Weight loss |
| **Testicular** | Painless lump/swelling in testis | Pain in testis |
|  |  | Anaemia |
|  |  | Anorexia |
|  |  | Fatigue |
|  |  | Weight loss |
| **Head and Neck** | Cervical lymphadenopathy | Dysphagia |
|  | Hoarseness | Anaemia |
|  | Lump | Anorexia |
|  | Sore throat | Fatigue |
|  | Stridor | Weight loss |
|  | Ulceration |  |
| **Leukaemia** | Bleeding | Anorexia |
|  | Bruising |  |
|  | Anaemia |  |
|  | Fatigue |  |
|  | Weight loss |  |
| **Lymphoma** | Bleeding | Anorexia |
|  | Bruising |  |
|  | Lump(s) |  |
|  | Night sweats |  |
|  | Pruritus |  |
|  | Anaemia |  |
|  | Fatigue |  |
|  | Weight loss |  |
| **Myeloma** | Bleeding | Anorexia |
|  | Bone pain |  |
|  | Bruising |  |
|  | Anaemia |  |
|  | Fatigue |  |
|  | Weight loss |  |
